# Uncertainty Calibration in Molecular Machine Learning: Comparing Evidential and Ensemble Approaches

**DOI:** 10.1002/chem.202503299

**Published:** 2026-02-04

**Authors:** Bidhan Chandra Garain, Max Pinheiro, Matheus de Oliveira Bispo, Mario Barbatti

**Affiliations:** ^1^ Aix Marseille University, CNRS, ICR Marseille France; ^2^ Institut Universitaire de France Paris France

**Keywords:** deep ensembles, machine learning, molecular simulation, post hoc calibration, uncertainty quantification

## Abstract

Machine learning (ML) models are increasingly used in quantum chemistry, but their reliability hinges on uncertainty quantification (UQ). In this study, we compare two prominent UQ paradigms—deep evidential regression (DER) and deep ensembles—on the QM9 and WS22 datasets, with a specific emphasis on the role of post hoc calibration. Raw uncertainties from both methods were systematically miscalibrated: DER produced uncertainty estimates where data noise and model uncertainty were not cleanly separated, while ensembles produced sharper yet underconfident estimates. Applying calibration techniques such as isotonic regression (ISR), standard scaling, and GP‐Normal corrected these deficiencies, aligning predicted variances with observed errors. On QM9, calibration enabled DER to filter high‐confidence predictions more effectively than ensembles. On WS22, calibrated ensembles not only improved statistical reliability but also delivered substantial computational savings in active learning, reducing redundant ab initio evaluations by more than 20%. These results demonstrate that post hoc calibration is essential to transform uncertainty estimates from descriptive metrics into actionable signals, ensuring both trustworthy predictions and resource‐efficient molecular modeling.

## Graphical abstract

Raw uncertainty estimates from deep evidential regression and deep ensembles are systematically miscalibrated. Post hoc calibration aligns predicted uncertainty with true errors, improving reliability and enabling efficient active learning and reducing computational cost while preserving predictive accuracy.

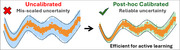

## 1 Introduction

Machine learning (ML) has become a powerful tool in chemistry, accelerating predictions and uncovering patterns beyond the reach of traditional simulations. Applications range from reaction mechanism modeling [1, 2, 3, 4], de novo generation of molecules [5, 6, 7, 8, 9], and force‐field development [10, 11, 12, 13, 14, 15], to navigating chemical space and predicting physicochemical properties [16]. These advances underscore the growing importance of data‐driven approaches in both fundamental and applied chemical research [17, 18, 19].

At their core, ML models are statistical tools. Their reliability depends not only on algorithmic design but also on how well the training data represent the underlying chemical space. When training datasets lack sufficient chemical or conformational diversity, predictions can become biased and uncertain, especially in out‐of‐distribution (OOD) regions [20]. Although historically the field emphasized increasing dataset size [21], recent studies show that scale without representativeness may reduce reliability [22]. This has led to a shift toward smart data strategies that prioritize smaller, well‐curated, and chemically diverse datasets tailored to the target prediction task [23, 24, 25].

These considerations are especially relevant in quantum chemistry, where generating reference data via high‐level electronic structure methods is computationally expensive and energy‐intensive. This raises both environmental concerns and barriers for researchers with limited computational access [26, 27, 28]. As a result, model effectiveness is now seen to depend as much on training data strategy as on model architecture [29, 30, 31]. Recent efforts emphasize datasets that are not only smaller but also physically informative and minimally redundant, capturing key chemical patterns [32, 33, 34]. Such strategies improve predictive accuracy while supporting more efficient and sustainable workflows [35, 36]. Still, the specific role of data selection in determining prediction reliability remains insufficiently explored [33]. Even well‐designed datasets may not fully address generalization challenges, especially in underrepresented regions.

This is where uncertainty quantification (UQ) becomes essential [37, 38, 39]. UQ enables the estimation of predictive confidence and identifies areas of chemical space where models are poorly supported. In doing so, it guides active learning [40, 41, 42] by prioritizing data points most likely to improve model performance. Since optimal training data vary depending on the predicted property, UQ is particularly useful for demanding tasks such as excited states, transition states, and nonadiabatic couplings. Its integration into ML workflows thus enhances both interpretability and efficiency.

Among UQ strategies, ensemble methods—where multiple independently trained models provide both mean predictions and predictive variance—are among the most widely adopted [43, 44, 45, 46]. Despite their effectiveness, training full ensembles can be costly, especially when using expensive quantum chemical labels. Efficient ensemble variants have been proposed to reduce this overhead. For instance, Kellner and Ceriotti introduced shared‐weight architectures to enable cost‐effective downstream uncertainty propagation [47]. Meanwhile, Bilbrey et al. combined readout ensembling and quantile regression to separately estimate epistemic and aleatoric uncertainties [48].

Other UQ methods offer complementary tradeoffs. Gaussian process regression (GPR) provides fully probabilistic estimates but scales poorly with dataset size [49, 50, 51]. Monte Carlo Dropout approximates Bayesian uncertainty using stochastic inference [52, 53]. More recently, evidential learning has emerged as a computationally efficient alternative [54]. Rather than relying on sampling, it models a higher‐order distribution over likelihood parameters, allowing for fast, single‐pass uncertainty estimation and principled decomposition of uncertainty sources [54, 55].

Regardless of the approach, meaningful uncertainty estimates require proper calibration [56, 57]. Calibration ensures that the predicted confidence levels correspond to actual prediction errors; without it, uncertainty values may become overconfident or underconfident [58, 59]. This is especially critical for applications such as property prediction and active learning, where model confidence directly guides data selection. Recent work has also explored fidelity‐informed strategies, in which lower‐cost quantum‐chemical methods guide sample selection. For instance, Vinod and Zaspel introduced LoUQAL, a low‐fidelity informed UQ approach that leverages inexpensive fidelities to improve active learning efficiency across diverse properties, while also demonstrating well‐calibrated uncertainty estimates [60]. Prior work, including Busk et al., has shown the value of recalibration for ensemble models [61]. However, calibration remains underutilized in other UQ paradigms. For example, Vazquez–Salazar et al. applied deep evidential regression (DER) to the QM9 dataset [55]. They later extended it to reactive PESs [62]—in both cases, without post hoc calibration to match uncertainties with observed errors.

Evaluating calibration involves comparing predicted uncertainties with actual error distributions. A common assumption in regression is that errors follow a normal distribution, enabling statistical prediction intervals via the Central Limit Theorem [63, 64]. Calibration curves and reliability diagrams assess coverage quality across the dataset [58], while *z*‐score analysis can reveal deeper variance‐related issues [57, 65, 66].

Despite progress in UQ development, standard training and inference pipelines often yield miscalibrated models [45, 47, 55, 61, 62, 67]. This study systematically examines how post hoc recalibration affects uncertainty estimates in molecular ML. We compare two contrasting paradigms: deep ensembles and DER. For scalar property prediction, we use QM9 [29], a benchmark of equilibrium geometries for small organic molecules—ideal for assessing global molecular property predictions such as electronic spatial extent. For geometry‐dependent energies and forces, we use WS22 [68], which provides nonequilibrium conformations and high‐quality PESs across a broad configurational range, enabling evaluation under OOD conditions.

Deep ensembles are widely adopted in molecular ML due to their simplicity and strong empirical performance [69]. DER, by contrast, offers a practical alternative to sampling‐based Bayesian methods. Traditional Bayesian approaches often rely on Markov Chain Monte Carlo (MCMC) or variational inference, which can struggle with convergence and scale [3, 54, 55]. DER bypasses this by learning evidential distributions in a single forward pass. Moreover, it provides principled decomposition into epistemic and aleatoric uncertainty, aligning well with our goal of dissecting these components in molecular systems.

In contrast to previous studies, which emphasized uncertainty estimation, our work integrates post hoc calibration techniques to improve reliability. We apply both parametric (e.g., GP‐Normal) and nonparametric (e.g., isotonic regression (ISR), standard scaling) methods to recalibrate uncertainty outputs from both DER and ensemble models. While other UQ methods exist, our focus is on comparing these two fundamentally distinct yet practical methods, which strike a balance between diversity, scalability, and relevance to the molecular ML community. We demonstrate that post hoc calibration improves their statistical alignment between uncertainty and error, while also offering benefits in downstream applications such as active learning—a dimension often overlooked in prior DER studies.

For QM9, we modified the final layer of an E(n)‐equivariant graph neural network (EGNN) [70, 71] to incorporate a DER output head for predicting uncertainty in electronic spatial extent. We also trained deep ensembles of unmodified EGNNs for comparison. For WS22, we adopted ensemble‐based models to study uncertainty calibration in extended PESs and force predictions.

Extending evidential methods to force predictions and energy‐conserving ML potentials is an active, promising direction [72]. However, doing this correctly requires additional architectural and algorithmic developments—such as energy/force‐consistent model architectures, careful treatment of vectorial force targets, and balanced energy–force loss weighting—which together warrant a dedicated methodological study. For this reason, we restrict the present work to calibrated uncertainties for scalar energies and force norms using ensemble baselines, leaving a full investigation of force‐consistent evidential models to future work. This allows us to assess calibration impact without introducing additional modeling complexity.

## 2 Methodology

### 2.1 E(n)‐Equivariant Graph Neural Network–Deep Evidential Regression

For predicting the electronic spatial extent in QM9, we used an EGNN as the regression model [71]. To quantify uncertainty, we extended the output layer with DER following Amini et al. [54] DER predicts the parameters (*γ*,*ν*,*α*,*β*) of a Normal–Inverse–Gamma (NIG) distribution, which defines the predictive mean and variance. This enables decomposition of uncertainty into an aleatoric component, reflecting data noise, and an epistemic component, reflecting model uncertainty. Training employed the evidential loss, which combines a negative log‐likelihood term with a regularization that discourages unjustified variance. Full mathematical derivations, additional equations, and hyperparameter details are available in the Supporting Information (Section: S1). The construction and training setup for the deep ensemble of EGNN models is described separately in Supporting Information (Section: S2).

### 2.2 ANI Neural Network Potential

To predict the uncertainty associated with an ML potential, we have utilized deep ensembles of ANI (a short for accurate neural network engine for molecular energies) models [14, 44, 73]. We systematically varied three hyperparameters across the ensemble: the width of the third hidden layer (32 or 64 neurons), the activation function (ReLU or CELU), and the learning rate (0.001 or 0.002). This design yielded eight distinct neural networks, each trained with a unique combination of these settings, thereby introducing epistemic diversity through controlled architectural and training‐level variations. In this work, we adopted an architecture‐diverse ensemble primarily to encourage decorrelated epistemic behavior. We note, however, that post hoc calibration operates solely on the joint relationship between predicted variances and empirical errors. As a result, calibrated uncertainties should exhibit the same qualitative behavior for ensembles composed of identical architectures trained with different random seeds or slightly perturbed training sets, which is the more common practice in ML force‐field development. Therefore, our conclusions regarding raw miscalibration and the benefits of recalibration are expected to hold across both ensemble constructions. The standard ANI formalism—including the decomposition of total energy into atomic contributions, derivation of forces as energy gradients, and the combined energy–force loss function—is provided in Supporting Information (Section: S3).

### 2.3 Uncertainty Quantification

To evaluate the calibration of uncertainty estimates in regression, we employed metrics specifically designed to assess how well predicted confidence levels correspond to actual prediction errors. The primary tools used were the average miscalibration curve and the corresponding calibration error, which quantify the deviation between predicted uncertainty intervals and the empirical distribution of errors. These metrics offer a focused evaluation of calibration quality, independent of the predicted mean. As a complementary measure, we computed sharpness [61], which captures the concentration of predictive distributions without reference to the ground truth. A model that assigns lower variance to its predictions is considered sharper. However, sharpness must be interpreted alongside calibration, since overconfident but miscalibrated models can be misleading despite their low variance.

To probe calibration robustness beyond average behavior, we further employed adversarial group calibration (AGC), a recently proposed method by Zhao et al. [74]. This approach evaluates not only overall calibration but also how well a model remains calibrated within subsets of the data that correspond to rare, heterogeneous, or difficult regions. For a given group size sε(0,1], defined as the fraction of the test set contained in a subgroup, AGC searches over all such subgroups and returns the calibration error of the worst (most miscalibrated) subgroup of that size. For example, a group size of 0.1 corresponds to the most miscalibrated 10% of the data, while larger group sizes reflect performance over increasingly broad regions of the input space. This provides a stringent and distribution‐aware measure of robustness, particularly in settings with limited coverage or nonuniform label distributions.

### 2.4 Post Hoc Calibration

Since both DER and deep ensembles produced systematically miscalibrated uncertainty estimates, we applied post hoc calibration methods to align predicted variances with empirically observed errors. Post hoc calibration operates on a held‐out calibration split: for each trained model, we freeze the network, predict on the calibration set, fit a recalibration mapping from predicted uncertainties to empirical errors, and then apply this mapping to all subsequent predictions. This procedure adjusts only the predicted uncertainties, leaving the underlying model unchanged, making it a flexible, architecture‐independent approach. We employed three complementary methods spanning nonparametric and parametric families [58, 75, 76].

- ISR: ISR [58] learns a monotone mapping from the model's raw uncertainty outputs on the calibration set (predicted standard deviations or nominal coverages) to empirical error levels. It makes no parametric assumption beyond monotonicity, so it can flexibly adjust the scale of predicted uncertainties while preserving their ranking. This allows systematic under‐ or over‐confidence to be corrected without changing the ordering of predictions.
- Standard scaling: Standard scaling [75] is a simple parametric recalibration that rescales all predicted standard deviations by a single global factor. This factor is learned on the calibration set so that the scaled uncertainties match the empirical error scale (for example, by minimizing the miscalibration area). The procedure preserves the Gaussian form and the relative ranking of uncertainties while correcting overall over‐ or under‐confidence.
- GP‐Normal: GP‐Normal [76] extends temperature scaling by learning an input‐dependent rescaling of the predictive variance with a Gaussian Process, while keeping the predictive mean fixed. The GP is trained on the calibration set so that the calibrated variances match the observed squared residuals. This yields a smooth, Bayesian mapping from input features to variance that can correct miscalibration in a flexible yet still parametric way.

All three methods require only a single additional fit on the calibration split and no retraining of the base model, which makes them well‐suited as post hoc baselines in our comparative study.

To ensure a broad and balanced evaluation, we combine GP‐Normal with both ISR and standard scaling. These methods span a range of assumptions and model capacities, from flexible parametric approaches to nonparametric baselines. This variety enables a systematic analysis of how different recalibration strategies affect the trustworthiness of uncertainty estimates.

These methods require no retraining, are computationally efficient, and span a range of assumptions—from flexible nonparametric fits to structured parametric corrections. Full algorithmic details are given in Supporting Information (Section: S4).

For nonparametric post hoc calibration, we utilized the uncertainty‐toolbox library [77], while for parametric post hoc calibration, we employed the netcal library [78]. All neural network training and inference were performed using PyTorch v2.3.1 with CUDA 12.1 support to leverage GPU acceleration. For the ANI model training and evaluation, we used the MLatom v3.9.1 package [79].

## 3 Results and Discussion

### 3.1 Predictive Uncertainty of EGNN–DER vs. Deep Ensembles of EGNN on QM9 Dataset

We utilized both the EGNN–DER model and deep ensembles of EGNNs to estimate uncertainty in predicting the electronic spatial extent from the QM9 dataset [29]. Considered a benchmark for molecular ML, QM9 consists of ∼130,000 equilibrium geometries of small organic molecules with quantum chemical properties computed at the B3LYP/6‐31G(2df,p) level. For the ensemble approach, eight independently initialized networks were trained with varied hyperparameters to promote diversity among models; the full configurations and corresponding mean absolute errors (MAEs) are reported in Supporting Information (Section: S2). The MAE of the EGNN–DER model is shown in Figure S1 and Supporting Information (Section: S5).

**FIGURE 1:**
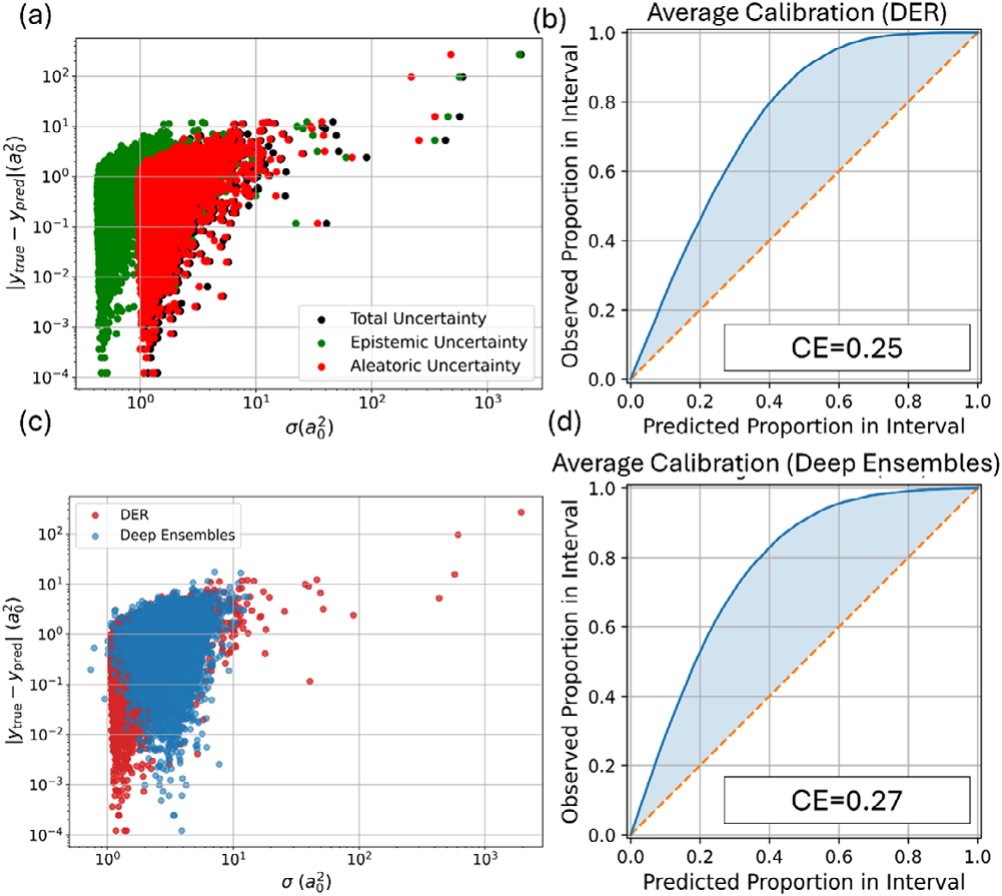
(a) Absolute error as a function of standard deviation coming from DER with its components (aleatoric and epistemic), (b) average miscalibration plot for the total uncertainty of DER, (c) comparison of the uncertainty arising from DER and deep ensembles, and (d) average miscalibration plot for the total uncertainty of deep ensembles. CE—calibration error.

We first examined the raw uncertainty estimates from both methods using absolute‐error plots, average calibration curves, and sharpness metrics. As shown in Figure 1a, EGNN–DER assigns significantly higher aleatoric than epistemic uncertainty, despite the deterministic nature of QM9, where all target values are derived from fixed DFT protocols without stochastic variation. This behavior stems from the structure of the evidential loss used to train DER. The NIG prior parameterizes a joint distribution over the predictive mean and variance, and the loss encourages broad predictive distributions in regions with large residuals to avoid overconfident errors. Because the model receives no supervision, separating epistemic from aleatoric components, the optimization can satisfy this penalty by increasing the aleatoric term, even when no intrinsic label noise is present.

Earlier analyses of evidential regression models have highlighted that this NIG‐based formulation is overparameterized and that the second‐order loss does not uniquely decompose total variance into “epistemic” and “aleatoric” channels, especially in low‐noise regimes [80]. In such settings, the evidence allocated to aleatoric and epistemic channels is not uniquely identifiable, and part of the model‐uncertainty signal is absorbed into the aleatoric variance. We therefore clarify that the elevated aleatoric component observed here is not indicative of physical noise in the QM9 labels, but a limitation of the standard DER formulation when applied to virtually noiseless quantum‐chemical datasets. Our analysis focuses on the calibrated total predictive variance, which remains meaningful even when the internal decomposition is not.

Figure 1c compares predicted uncertainties with absolute errors for both models. Both show a positive correlation, confirming that uncertainty estimates carry predictive value. However, DER outputs a broader range of uncertainty values, with a high sharpness score of 19.05 (Table 1), indicating wide and less discriminative intervals. Deep ensembles, by contrast, exhibit tighter uncertainty distributions with a sharpness of 3.28—allowing for finer separation between low‐ and high‐error predictions. This sharper ranking is beneficial for applications such as outlier detection or prioritizing predictions in conservative pipelines.

**TABLE 1:** Summary of uncertainty quantification metrics for EGNN–DER and deep ensembles.

Metric	DER	Deep ensembles	Better method
Calibration error (CE)	0.25	0.27	DER
Sharpness (lower is better)	19.05	3.28	Deep ensembles

Figure 1b, d shows the average miscalibration curves. Both methods were initially underconfident, with DER slightly outperforming ensembles in calibration error (0.25 vs. 0.27). However, DER's broad intervals—while better aligned on average—lacked the precision needed to isolate high‐confidence predictions in practice.

**FIGURE 2:**
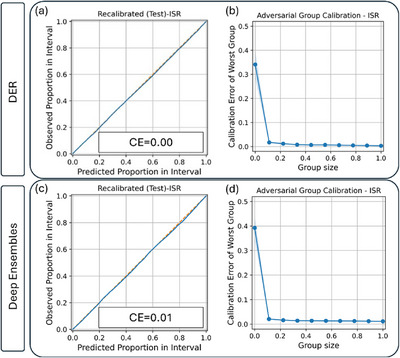
(a) Average miscalibration plot for the test dataset after post hoc calibration with isotonic regression for DER derived uncertainty, (b) adversarial group calibration plot of post hoc calibrated DER derived uncertainty, (c) average miscalibration plot for the test dataset after post hoc calibration with isotonic regression for deep ensembles derived uncertainty, and (d) adversarial group calibration plot of post hoc calibrated deep ensembles derived uncertainty.

To improve reliability, we applied ISR as a post hoc recalibration method. As shown in Figure 2a, c, this reduced CE to 0.00 for DER and 0.01 for ensembles, indicating excellent average calibration. To evaluate robustness across different parts of the input space, we employed AGC, which measures the worst‐case deviation over randomly drawn subsets. As defined earlier, the group size represents the fraction of the test set used to form a subgroup, and the AGC curve reports the calibration error of the worst subgroup at each size. Figure 2b, d show that both models achieved low AGC error after recalibration, with DER demonstrating greater consistency across heterogeneous subpopulations. We also applied standard scaling and GP‐Normal calibration (Figure S2 of (Section: S5)), both of which yielded similarly low CE and AGC values, further confirming the robustness of post hoc calibration across transformations.

While CE and AGC reflect statistical alignment, they do not directly inform downstream utility. We therefore applied a confidence‐based filtering analysis, using the criterion 2*σ* < 1$a_{0}^{2}$ to select high‐confidence predictions. This threshold is entirely heuristic and was chosen to define a narrow predictive band—small enough to reflect high certainty, yet broad enough to capture a meaningful number of predictions. No active learning simulation was performed; instead, this test serves as a proxy to examine how well the model can confidently identify correct predictions based on its calibrated uncertainty. As such, the filtering analysis provides a necessary but not sufficient condition for evaluating suitability in uncertainty‐guided selection. Whether this advantage holds in an iterative active learning loop remains an open question for future study.

To quantify the effect of calibration on confidence‐based filtering, we evaluated the criterion 2σ<1 (in units of $a_{0}^{2}$) using both the raw and post‐calibrated uncertainties. Before calibration, no test samples satisfied this condition (0 of 13,083), reflecting that the raw uncertainties were severely mis‐scaled.

After applying the ISR calibration, 3307 test samples satisfied $2\sigma_{\mathrm{ISR}}<1$. Based on the true error condition $|y_{\mathrm{true}}-y_{\mathrm{pred}}|<1$, 11,461 samples in the test set qualify as truly “confident.” Among these are as follows:

- 3221 were correctly retrieved by the model (True–True).
- 8240 were truly confident but not selected (True–False, i.e., missed).

Among the 3307 samples selected as confident, only 86 had errors ≥ 1 (False–Positive, i.e., overconfident).

Thus, calibration enables the model to recover a meaningful confident subset (3307 samples), whereas the uncalibrated model identifies none, and the selected subset remains largely reliable (False–Positive rate ≈ 2.6%).

These results show that calibration not only rescales uncertainty magnitudes but also enables the model to meaningfully separate a small but high‐precision subset of confident predictions—something the uncalibrated model cannot achieve at all. The calibrated confident subset exhibits very low error contamination (86/3307 3307 ≈ 2.6%). Ensembles, though sharper, selected only 37 predictions within the same confidence bound, suggesting their post‐calibrated uncertainty distribution remained too narrow for practical triage.

This comparison highlights a fundamental tradeoff. Deep ensembles produce inherently sharper and more discriminative uncertainty estimates, which are advantageous for ranking or threshold‐based decision‐making. However, DER's broader dynamic range—although imprecise in its raw form—benefits more from recalibration, enabling decisive filtering of high‐confidence predictions in practice. This is advantageous in tasks such as active learning or dataset curation, where confidently discarding or prioritizing samples can reduce labeling cost or computational burden.

Finally, DER offers this flexibility using a single model, whereas ensemble methods require training multiple networks—an important consideration when computational resources are constrained. These findings underscore that UQ must be paired with robust calibration to be effective in real‐world molecular ML applications.

### 3.2 Predictive Uncertainty of Deep Ensembles on WS22 Acrolein Dataset

The WS22 dataset is a recent benchmark built from molecular geometries generated via harmonic‐oscillator Wigner sampling for several molecules and geodesic interpolation between *trans* and *cis* conformations for some species [68]. It covers a broad configurational space, making OOD predictions particularly challenging. Energies and forces for the dataset were computed at the PBE0/6–311G* level of theory. The acrolein subset contains 120,000 geometries: 50,000 in the *trans* conformation, 50,000 in the *cis* conformation, and 20,000 obtained from geodesic interpolation between them. For model training, we used only the *trans* conformers and applied ANI deep ensembles with a 90:10 train–validation split. In this experiment, rather than reproducing a typical active‐learning scenario with a limited training set, the goal was to specifically probe uncertainty calibration in an extrapolative regime, where we prioritized a highly accurate *trans*‐region model before evaluating its performance on the distinct *cis* geometry distribution. To ensure that the model achieved reliable force accuracy within the *trans* manifold, we used 90% of the *trans* configurations for training and reserved the remaining 10% for testing. Although the state‐of‐the‐art equivariant models can often reach sub‐kcal/mol accuracy with only a few hundred configurations, our choice of a larger training fraction was intentional: it reduces confounding effects from poor coverage of the trans region. It allows us to isolate the impact of extrapolation toward *cis*‐like geometries on the resulting uncertainty estimates.

Our approach combines deep ensembles for UQ with a Query‐By‐Committee (QBC) strategy for active learning. The ensemble's prediction variance quantifies epistemic uncertainty, guiding data selection and iteratively improving model generalization in active learning. We evaluated the predictive uncertainties on the geodesic interpolation points, which represent a distribution shift. This setup mimics realistic active‐learning scenarios, especially in surface hopping dynamics [81, 82, 83].

We generated an ensemble of eight ANI models by varying three hyperparameters. The variations included the activation function (ReLU or CELU), learning rate (0.001 or 0.002), and the number of neurons in the third layer (32 or 64). All models achieved accuracy within 1 kcal/mol, meeting the threshold for chemical accuracy. The hyperparameter settings and the corresponding performance for energy and forces are reported in Table S3. The other hyperparameters are shown in Table S4. Among the tested options, CELU consistently outperformed ReLU, producing lower RMSE values for both energy and force predictions.

**FIGURE 3:**
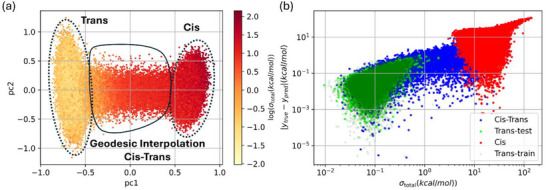
(a) 2D projection of the WS22 dataset onto the first two principal components (PC1 and PC2), with points colored by the log of the predicted total energy uncertainty (*σ*_total_) in kcal/mol, (b) represents the relationship between the total uncertainty (*σ*_total_​), and the absolute prediction error (∣*y*_true_−*y*_pred_∣) in kcal/mol.

Figure 3a shows a 2D projection of the WS22 dataset onto the first two principal components (PC1 and PC2). Points are colored by the log of the predicted total energy uncertainty (*σ*_total_​) in kcal/mol. The *trans* conformers occupy a low‐uncertainty region, reflecting the model's confidence. This is expected, since 90% of the *trans* conformers were included in the training set. In contrast, the *cis* conformers and the geodesic interpolation points exhibit significantly higher uncertainties, indicating a distribution shift between the training and test datasets. This trend reflects the model's difficulty in underrepresented regions.

Figure 3b represents the relationship between the total energy uncertainty (*σ*_total_​) in kcal/mol and the absolute prediction error (∣*y*_true_−*y*_pred_∣) on a logarithmic scale. Data points are categorized as *trans*‐train (dark green), *trans*‐test (light green), *cis* (red), and *cis*–*trans* interpolation (blue). The strong positive correlation between *σ*_total_ ​ and the prediction error (∣*y*_true_−*y*_pred_∣) highlights the reliability of the uncertainty predictions. The *trans* conformers (both training and test subsets) cluster in the low‐error, low‐uncertainty region, while the *cis* conformers and interpolation points dominate the high‐uncertainty regime. This behavior demonstrates that the model can identify low‐confidence regions, a crucial step for recognizing challenging configurations during active learning.

Separating high‐uncertainty points highlights their value for prioritizing future sampling in underrepresented regions of configurational space. To evaluate the quality of energy‐uncertainty predictions, we analyzed the average miscalibration curves for both the test set and the *cis*–*trans* interpolation region. Both show slight underconfidence, with calibration errors of 0.11 and 0.10, respectively. Post hoc calibration markedly improved these estimates, yielding low CE and AGC errors across multiple methods. For full calibration curves, adversarial metrics, and method comparisons, see Supporting Information (Section: S6).

Building on the energy uncertainty analysis, we next evaluated forces by quantifying the uncertainty in the norm of the force vector, derived from the energy gradient. Although simulations are sensitive to individual force components, we verified in Supporting Information (Section: S7) that the predicted force‐norm uncertainties correlate strongly with both (i) the angular disagreement between predicted and reference force vectors and (ii) per‐atom force errors along the *cis*–*trans* pathway. This indicates that the norm‐based uncertainties are a reasonable proxy for detecting regions where component‐wise forces are unreliable. This scalar measure provides a tractable input for UQ and serves as a valuable proxy for identifying regions with directional force errors.

**FIGURE 4:**
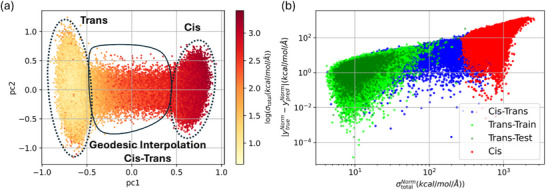
(a) 2D projection of the WS22 dataset onto the first two principal components (PC1 and PC2), with points colored by the log of the predicted total force uncertainty in kcal/mol/Å. (b) Relationship between the total force uncertainty ($\sigma_{\mathrm{total}}^{\mathrm{Norm}}$​) and the absolute prediction error $|y_{\mathrm{true}}^{\mathrm{Norm}}-y_{\mathrm{pred}}^{\mathrm{Norm}}|$ on a logarithmic scale. Data points are categorized into subsets: *trans*‐train (dark green), *trans*‐test (green), *cis* (red), and *cis*–*trans* interpolation (blue).

Figure 4a shows a similar 2D projection of the WS22 dataset onto the first two principal components, with points colored by the logarithm of the predicted total force uncertainty ($\sigma_{\mathrm{total}}^{\mathrm{Norm}}$​) in kcal/mol/Å. The *trans* conformers, dominating the training set (90%), exhibit low force uncertainties, highlighting the model's confidence in these well‐sampled regions. By contrast, *cis* conformers and geodesic interpolation points exhibit significantly higher uncertainties, reflecting their under‐representation in the training set.

Force uncertainties follow the same trends as energy uncertainties. Figure 4b shows a strong correlation between predicted uncertainty and actual error, confirming that the estimates are reliable. *Trans* conformers cluster in the low‐error, low‐uncertainty regime, while *cis* and interpolation points dominate the high‐error, high‐uncertainty region. This clear separation indicates that force uncertainty effectively identifies configurations that require additional sampling.

We note that Figures 3 and 4 display the raw predicted uncertainties, as they most clearly illustrate the qualitative trend of increasing uncertainty as one moves from the *trans* (training) region toward *cis* (unseen) and interpolated configurations. Post hoc calibration does not alter this qualitative structure: the separation between *trans*, *cis*–*trans*, and *cis* points remains essentially unchanged. Instead, calibration primarily adjusts the quantitative scale of the predicted uncertainties so that their magnitudes more accurately reflect the empirical absolute errors.

We assessed force uncertainty using miscalibration curves and AGC, comparing predicted uncertainties with actual errors for both the test set and the *cis*–*trans* interpolation region. Initial analysis revealed significant underconfidence in force‐uncertainty predictions, with calibration errors (CE) of 0.37 in both cases—markedly different from the better‐calibrated energy uncertainties. Post hoc calibration using ISR substantially reduced the CE to 0.01 and improved AGC performance, indicating both global and subgroup reliability. Standard scaling and GP‐Normal calibration produced similar improvements, though the scaling factor was relatively low (0.208) due to the strong initial underconfidence. For full analysis of force uncertainty calibration, including miscalibration and AGC plots before and after recalibration, see Supporting Information (Section: S8).

**FIGURE 5:**
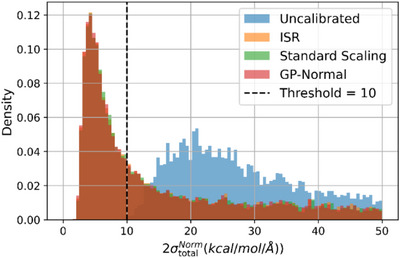
Histogram of normalized total force uncertainties for uncalibrated and post hoc‐calibrated models. The vertical dashed line denotes the 10 kcal/mol/Å uncertainty threshold used to determine whether a data point requires further training. The uncalibrated model (blue) exhibits a broad spread with most points exceeding the threshold, while post hoc methods (ISR, Standard scaling, GP‐Normal) shift the uncertainty distributions significantly to the left. This indicates that a much larger fraction of data can now be confidently retained without retraining, reducing redundant computations and improving active learning efficiency.

Table 2 and Figure 5 show that post hoc calibration reduces redundant computations. For energy predictions, the number of low‐uncertainty points (defined by 2*σ*_i_E < 1 kcal/mol) increases substantially following calibration. For example, the ISR method identifies 5904 such points, compared to just 5226 in the uncalibrated model, yielding over 670 redundant computations saved. The number of high‐error predictions among these low‐uncertainty points increases from 11 to 46, representing a more than 300% rise. Although this may seem unfavorable, it reveals that calibration exposes previously overconfident errors, bringing uncertainty estimates in line with actual performance.

**TABLE 2:** Post hoc recalibrated uncertainty comparison for energy and force predictions.

	Energy Threshold = 1 kcal/mol		Force norm Threshold = 10 kcal/mol/ **Å**	
Calibration method	Low uncertainty points (2$\sigma_{i}^{E}\;<$ Threshold)	$|y_{i}^{\mathrm{true}}-y_{i}^{\mathrm{pred}}|$> Threshold	Low uncertainty points (2$\sigma_{i}^{F}<$ Threshold)	$|y_{i}^{\mathrm{true}}-y_{i}^{\mathrm{pred}}|$ > Threshold
Uncalibrated	5226	11	3	0
ISR	5904	46	4546	61
Standard scaling	5875	44	4518	60
GP‐Normal	5945	48	4549	61

The improvements are even more striking for force predictions. In the uncalibrated case, only three data points fall below the force uncertainty threshold (2$\sigma_{i}^{F}<\;10\;\mathrm{kcal}/\mathrm{mol}/\;\;$ Å), meaning nearly all data would be flagged for retraining or additional evaluation. However, calibration with ISR boosts this number to 4546, while standard scaling and GP‐Normal yield similar gains. This translates to more than 4500 force evaluations avoided, significantly reducing computational cost. The number of incorrectly trusted points rises from 0 to about 61, but the calibrated models remain largely reliable.

**FIGURE 6:**
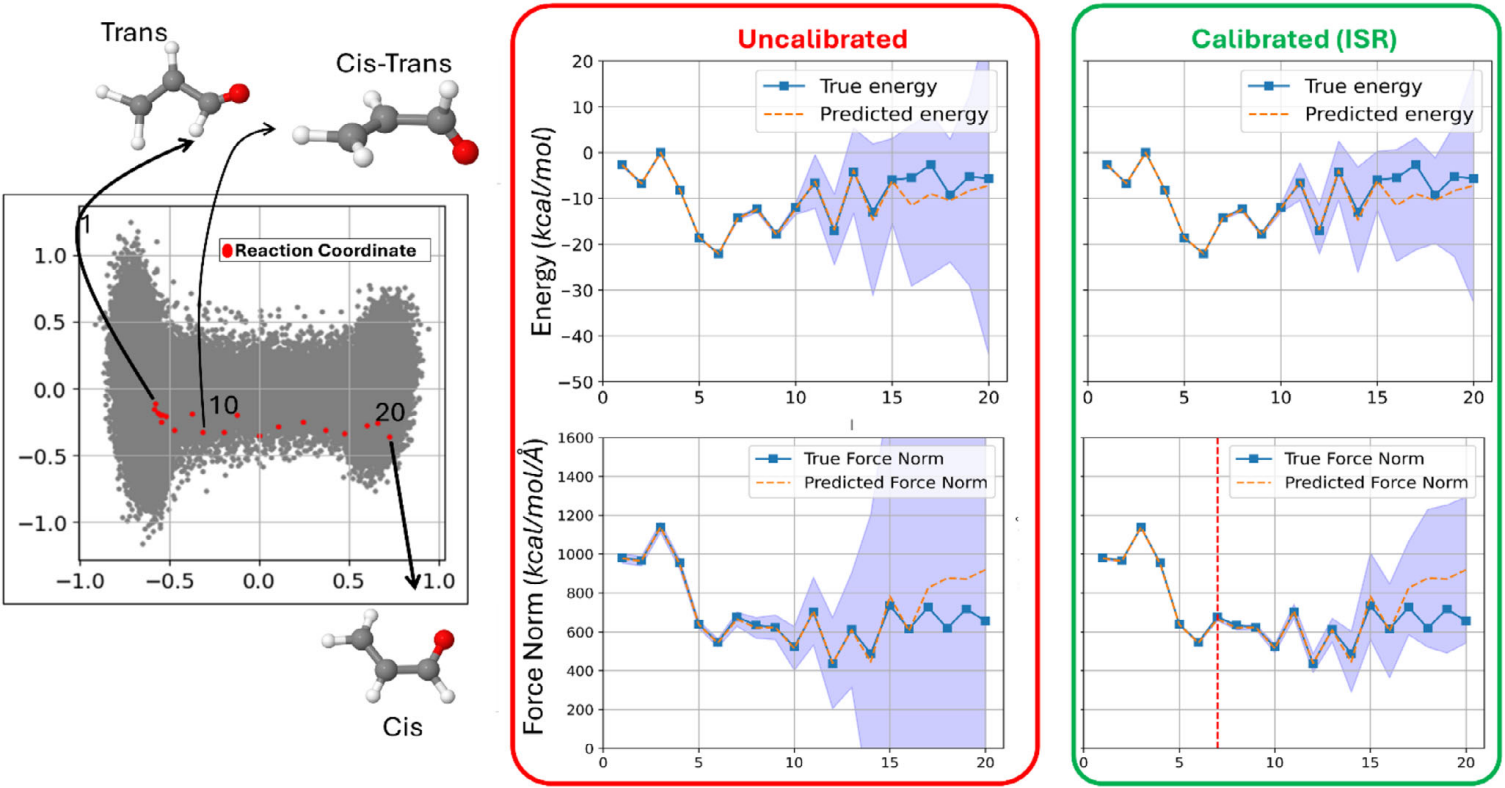
Effect of post hoc uncertainty calibration on active learning query strategy along the acrolein *cis*–*trans* isomerization path. (Left) Reaction coordinate (red) projected onto a low‐dimensional space constructed from the dataset. (Right) Comparison of predicted and true energy (top) and force norm (bottom) for 20 geometries along the pathway using uncalibrated (red box) and ISR‐calibrated (green box) uncertainty estimates. The shaded region denotes ±2*σ* predictive uncertainty.

Figure 6 illustrates the distribution of normalized total force uncertainties before and after applying post hoc calibration. The uncalibrated model exhibits a broad, right‐skewed distribution, with most points exceeding the active learning threshold of 10 kcal/mol/Å, suggesting that nearly the entire dataset would be flagged for further training. This indiscriminate flagging reflects a lack of trustworthiness in the model's uncertainty estimates, undermining the efficiency of active learning. Post hoc calibration—especially ISR—significantly narrows and shifts this distribution to the left, bringing a substantial fraction of points below the threshold. This recalibration produces a clearer separation between high‐ and low‐confidence predictions, allowing more targeted sampling and reducing computational redundancy. For instance, ISR increases the number of low‐uncertainty points by more than 4500, resulting in over 23% fewer ab initio force evaluations. Although high‐error low‐uncertainty predictions rise from 0 to 61, this tradeoff is acceptable given the reduced query load. Overall, post hoc calibration not only corrects statistical miscalibration but also makes uncertainties more useful for downstream tasks, such as active learning.

To further investigate the practical benefits of post hoc calibration, we applied it within an active learning context where molecular geometries along a reaction coordinate are already available. Specifically, we analyzed how force uncertainty calibration influences the *query strategy*—the selection of new data points for high‐fidelity evaluations—during conformational exploration. As shown in Figure 6, we evaluated both uncalibrated and ISR‐calibrated models on a transition path between *cis* and *trans* isomers of acrolein.

Calibration does not alter the predicted means or the ranking of structures by error; it adjusts the uncertainty estimates to match the empirical error distribution. This makes uncertainty a more reliable indicator of predictive confidence, which is critical for active learning, error‐aware sampling, and model refinement. In this case, energy uncertainties were already well calibrated, so we focused on force norm uncertainty. Figure 6 (left) shows the projection of the reaction pathway (in red) in the latent (PCA) space. The right panels compare predicted vs. true energy and force norms across the 20 geometries sampled along this reaction coordinate.

In the uncalibrated model (red panel), the force uncertainties remain high throughout the entire trajectory, as evidenced by the wide uncertainty bands. Consequently, every point along the reaction path would be considered uncertain, triggering costly *ab initio* quantum calculations even when they might not be necessary. In contrast, after ISR‐based calibration (green panel), the uncertainty bands contract significantly for the initial segment of the trajectory. The vertical dashed red line marks Point 7, the last geometry for which the total force‐norm uncertainty remains below the active‐learning threshold of 10 kcal/mol/Å. This delineates a natural cutoff: points to the left of this threshold now fall below the uncertainty requirement (2$\sigma_{i}^{F}$< 10 kcal/mol/ Å), and thus do not require retraining or high‐level calculations. The same strategy was applied to nine other reaction pathways in Supporting Information (Section: S9), showing consistent improvements in query efficiency after calibration.

This change has major implications for query efficiency: without calibration, active learning would start querying from the very beginning of the reaction path, leading to redundant evaluations. With calibration, the first 7 points can be confidently skipped, as their low uncertainty makes retraining unnecessary. In this way, post hoc calibration converts uncertainty from a descriptive statistic into a practical signal for targeted sampling, allowing active learning to focus on genuinely ambiguous configurations and reducing computational costs.

**FIGURE 7:**
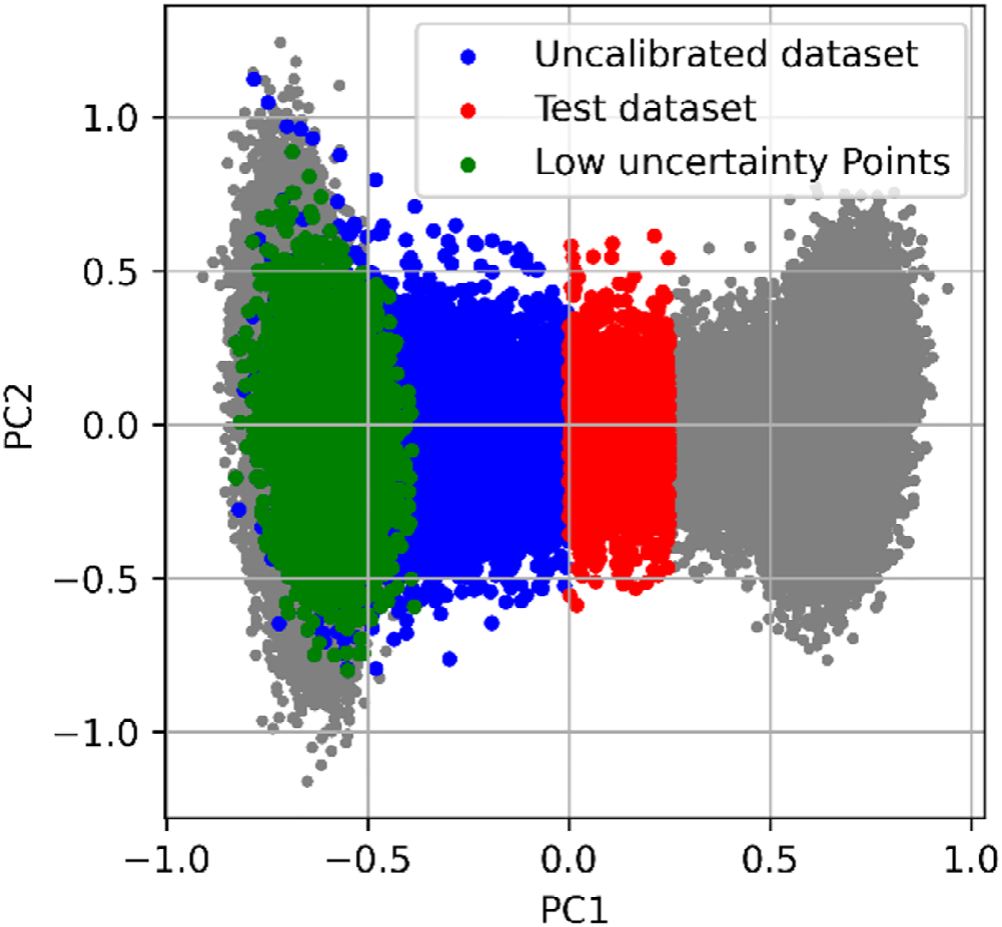
Comparison of uncalibrated and ISR‐calibrated uncertainty‐based data selection strategies for active learning. A pretrained model on trans conformations was evaluated on an out‐of‐distribution (OOD) region (red). Training with the full high‐uncertainty region (blue) yields similar accuracy to training with only the ISR‐selected subset (blue minus green), which excludes low‐uncertainty points (green).

To evaluate the effectiveness of ISR‐calibrated uncertainty for active learning, we used a pretrained model trained on 45,000 trans conformations. We tested its performance on a distinct OOD region in the PCA space (shown in red) in Figure 7. This red region does not overlap with the training distribution (left side) nor with the low‐uncertainty region (green); it therefore represents a realistic and challenging test case for generalization.

We then selected new training points from a high‐uncertainty region (blue) based on two strategies:

- Uncalibrated selection: All points in the blue region with uncertainty > 10 kcal/mol/Å were selected, regardless of prediction reliability.
- ISR‐calibrated selection: From the same blue region, we excluded the green points identified by ISR‐calibrated uncertainty as already reliable, thereby avoiding redundant training. The pretrained model already achieves strong performance on these low‐uncertainty points, with energy RMSE of 0.13 kcal/mol and a force RMSE of 0.62 kcal/mol/Å, indicating that additional training on them is unnecessary.

This experiment simulates a single active learning iteration, in which uncertainty‐guided selection triggers targeted retraining from an existing model. By keeping the model architecture and retraining procedure constant, the setup isolates the effect of uncertainty calibration on data selection efficiency, without interference from other active learning hyperparameters.

We trained the updated models on the selected points and evaluated them on the same red OOD region. To ensure a fair comparison, both models were fine‐tuned from the same pretrained base and evaluated on the same OOD test region, thereby guaranteeing consistent performance metrics. Results (Table 3) show that the ISR‐calibrated strategy achieves comparable performance with significantly fewer training points.

**TABLE 3:** Performance comparison with and without calibrated uncertainty‐based data selection. The ISR‐calibrated strategy uses 4546 fewer training points than the uncalibrated approach while achieving similar accuracy on an out‐of‐distribution test set, demonstrating improved sampling efficiency.

Training dataset	Model	Points used for training	Energy RMSE (kcal/mol)	Force RMSE (10 kcal/mol/Å)
Trans region	Pretrained model	45,000	3.03	9.68
Trans region + uncalibrated selection (blue region)	Model trained on uncalibrated sampling	45,000 + 10,045	0.26	1.26
Trans region + calibrated selection (blue–green region)	Model trained on calibrated sampling	45,000 + 5,499	0.23	1.20

The ISR‐calibrated strategy achieves comparable accuracy while using 4546 fewer training points. This corresponds to a 45.3% reduction relative to the high‐uncertainty pool considered for selection (blue region), even though it represents an 8.2% reduction relative to the full 45,000‐point dataset. This demonstrates that calibration prevents unnecessary resampling of points that the pretrained model already predicts reliably. Although we focus on a single‐loop analysis, extending this strategy to multi‐round active learning is a natural next step, particularly for iterative workflows such as adaptive sampling in molecular dynamics or excited‐state dynamics. In summary, post hoc calibration makes uncertainty estimates both reliable and cost‐saving, enabling efficient exploration of configurational space.

## 4 Conclusions

This work highlights the central role of post hoc calibration in making UQ reliable and actionable in molecular ML. Using QM9 and WS22 as benchmarks, we compared DER and deep ensembles, showing that raw uncertainties from both methods were systematically miscalibrated.

For QM9, DER tended to overestimate aleatoric uncertainty while ensembles produced sharper but underconfident estimates. Post hoc calibration, especially ISR, effectively corrected these biases and transformed both approaches into trustworthy predictors, with DER benefiting most from recalibration in confidence‐based filtering.

For WS22, deep ensembles successfully identified distribution shifts but initially underestimated uncertainty for energies and forces. Calibration significantly improved alignment with true errors, yielding tangible efficiency gains in active learning and avoiding thousands of redundant quantum chemical evaluations.

Overall, our findings establish that post hoc calibration is not optional but essential: it bridges the gap between statistical uncertainty estimates and their practical use in sampling, error‐aware prediction, and data‐efficient exploration of chemical space.

## Author Contributions

Conceptualization: B.C.G. and M.B. Data curation: B.C.G. Formal analysis: B.C.G. Funding acquisition: M.B. Methodology: B.C.G. and M.P.J. Project administration: M.B. Supervision: M.B. Visualization: B.C.G. Writing – original draft: B.C.G. and M.B. Writing – review and editing: M.P.J. and M.O.B.

## Conflicts of Interest

The authors declare no conflicts of interest.

## Supporting information




**Supplementary File 1**: chem70741‐sup‐0001‐SuppMat.pdf.

## Data Availability

The code and data generated for this work are available at GitHub and Zenodo. The QM9 dataset was obtained from PyTorch Geometric, and the WS22 acrolein dataset is accessible at Zenodo.
