# Supplementary material for: Uncertainty Calibration in Molecular Machine Learning: Comparing Evidential and Ensemble Approaches
Source: Chemistry. 2026 Feb 4;32(15):e03299. doi: 10.1002/chem.202503299 (PMC13107503; doi:10.1002/chem.202503299)
Supplement: Supplementary file 1 — chem70741‐sup‐0001‐SuppMat.pdf. [file CHEM-32-e03299-s001.pdf]

Uncertainty Calibration in Molecular Machine Learning: Comparing Evidential and Ensemble Approaches

Bidhan Chandra Garain, Max Pinheiro Jr., Matheus de Oliveira Bispo, Mario Barbatti

Supporting Information

Contents

S 1. Deep Evidential Regression for Uncertainty Prediction 1
S 2. Deep Ensemble of EGNN 5
S 3. ANI models 6
S 4. Post hoc calibration techniques 8
S 5. Post hoc calibration results of EGNN-DER 10
S 6. Energy Uncertainty Calibration in WS22 12
S 7. Justification for Using Force Norm in Uncertainty Calibration 14
S 8. Calibration of Force Uncertainty Predictions 16
S 9. Force Uncertainty Thresholding Across Geodesic Paths 17
S 10. References 22

S 1. Deep Evidential Regression for Uncertainty Prediction

Evidential regression is a regression technique that aims to model not only the relationship between inputs and target variables but also their uncertainties. These uncertainties have two origins: epistemic, which comes from the model's shortcomings in describing the relationship, and aleatoric, which is inherent to the dataset, such as noise. In the context of Deep Learning, a DER model is a deep neural network capable of modeling a probability distribution given a set of training data, outputting the parameters of said distribution.

For predicting the electronic spatial extent of the QM9 dataset, we opted for an E(n)-equivariant graph neural network (EGNN) as the regression model.[1] The message-passing network with $l = 1,\ldots,$ L-1 layers is defined as follows: first, a graph analogous to the molecule's structure is constructed, in which nodes represent individual atomic nuclei and edges represent potential interactions within a predetermined cutoff radius. The initial node embedding from the atomic numbers Z_i,

$$h_i^0 = \psi_o(Z_i) \tag{1}$$

The message m_{ij}^l passed between node i and j is defined for layers $l = 0, \ldots, L-1$ as,

$$m_{ij}^l = \varphi_m\left(h_i^l, h_j^l, d_{ij}\right) \tag{2}$$

where d_{ij} denotes the Euclidean distance between node i and j,

$$d_{ij} = \left\|X_i - X_j\right\|^2 \tag{3}$$

The node embeddings are then updated using aggregated messages for layers $l = 0, \ldots, L-1$,

$$h_i^{l+1} = \varphi_h\left(h_i^l, \sum_{j\neq i} m_{ij}^l\right) \tag{4}$$

φ_m, φ_h are usually parametrized by multilayer perceptrons (MLPs) on messages and node updates.

After aggregating the node embeddings, we extend the network's output to include parameters that capture predictive uncertainty, following the setup previously used in the DER framework by Amini *et al.*[2] Specifically, the model predicts four parameters — γ (mean), ν (evidence), α (shape), and β (scale), which together define a Normal-Inverse-Gamma (NIG) distribution over the target variable. To model uncertainty in regression, we treat each prediction as a random variable drawn from a probabilistic distribution. In this framework, we assume a hierarchical

model where the target mean and variance are governed by an NIG prior. This choice is motivated by the NIG distribution's conjugacy to the Gaussian likelihood, enabling closed-form posterior updates.[2] Under this setup:

- The predictive mean μ follows a normal distribution with mean γ and precision scaled by the evidence ν.
- The predictive variance $σ^2$ is sampled from an inverse gamma distribution determined by α and β.

$$\mu \sim \mathcal{N}(\gamma, \sigma^2 \nu^{-1}), \sigma^2 \sim \Gamma^{-1}(\alpha, \beta)$$

$\Gamma^{-1}$is the inverse gamma distribution, and $\gamma \in \mathbb{R}, \nu > 0, \alpha > 1$ and $\beta > 0$. The model infers a posterior distribution over the predictive mean and variance.

This posterior is assumed to decompose into independent distributions for mean and variance, consistent with the properties of the NIG family. This framework allows the network to express both confidence and prediction spread without requiring sampling or ensembles,

$$q(\mu, \sigma^2) = g(\mu) f(\sigma^2) \tag{5}$$

While in general, the posterior over (μ, σ^2) is not factorized, in the NIG prior framework, it naturally factorizes into a Gaussian for the mean and inverse gamma for the variance, as part of the conjugate prior structure. This factorization is encoded in the NIG distribution, which allows us to obtain closed-form expressions for the total uncertainty and predictive variance.

Therefore, our approximation follows the structure of a Gaussian conjugate prior, represented explicitly by the NIG distribution,

$$q(\mu, \sigma^2 | \gamma, \nu, \alpha, \beta) = \frac{\beta^\alpha \sqrt{\nu}}{\Gamma(\alpha)\sqrt{2\pi\sigma^2}} \left(\frac{1}{\sigma^2}\right)^{\alpha+1} exp\left(-\frac{2\beta + \nu(\gamma - \mu)^2}{2\sigma^2}\right) \tag{6}$$

Given a NIG distribution, we can compute the property prediction as follows:

$$\mathbb{E}[\mu] = \gamma \tag{7}$$

To recapitulate, for each prediction, our DER model estimates parameters of a NIG distribution: mean γ, shape α, scale β, and evidence ν. From these, we decompose the predictive uncertainty as follows:

Aleatoric uncertainty is estimated as the expected data noise:

$$\mathbb{E}[\sigma^2] = \frac{\beta}{\alpha - 1} \tag{8}$$

This represents data uncertainty, which is the noise in the target variable that cannot be reduced even with more data (e.g., fluctuations or numerical noise from DFT calculations). Aleatoric uncertainty depends on α and β, which are outputs of the network and parameters of the inverse gamma distribution.

Epistemic uncertainty is estimated as the variance of the predictive mean:

$$Var[\mu] = \frac{\mathbb{E}[\sigma^2]}{\nu} \tag{9}$$

This represents model uncertainty, which is a measure of confidence in the predicted mean. This term shrinks with more evidence (higher ν or α) and grows when data is scarce. These quantities are estimated directly from the network's outputs, enabling single-pass UQ without ensembles. Related applications to molecular properties have been discussed by Vazquez *et al.*, though without the post hoc calibration techniques employed in our work.[3] The hyperparameters used in the training are given in Table S1.

The evidential loss function comprises two components: the first is the negative log-likelihood (NLL) loss, which ensures accurate prediction of both mean and variance; the second is a regularization term, scaled by a coefficient λ, which penalizes unjustified uncertainty by discouraging the model from predicting high variance in the absence of high residual errors.

$$\mathcal{L}_i(\boldsymbol{w}) = \mathcal{L}_i^{NLL}(\boldsymbol{w}) + \lambda\mathcal{L}_i^{reg}(\boldsymbol{w}) \tag{10}$$

where $\mathcal{L}_i^{reg} = |y_i - \gamma|(2\nu + \alpha)$.

Table S1: EGNN-DER model and training hyperparameters

**Model Parameters**	**Value**
Hidden channels	64
Number of message passing layers	3
Activation Function	SiLU
Normalization mode	Batch Normalization
Pooling method	Global Mean Pooling
**Training parameters**	**Value**
Loss function	Evidential regression loss
Regularization coefficient (λ)	10^{-2}
Optimizer	Adam
Learning rate	2×10^{-4}
Batch size	32
Weight decay	1×10^{-8}

Epochs	100

S 2. Deep Ensemble of EGNN

To construct the ensemble of EGNNs, eight independently initialized networks were trained with varying hyperparameters, including hidden dimensions, message-passing layers, and activation functions. The complete configuration details, along with their mean absolute errors (MAE), are provided in Table S2.

Table S2: Summary of deep ensembles of EGNN

Model No.	Hidden channels	Number of message passing layers	Activation function	MAE
1	64	3	SiLU	3.23
2	128	3	SiLU	2.43
3	128	4	SiLU	2.35
4	64	4	SiLU	1.47
5	64	3	ReLU	3.75
6	128	3	ReLU	5.15
7	128	4	ReLU	2.17
8	64	4	ReLU	2.06

S 3. ANI models

The ANI model establishes a relationship between the molecular geometry, represented as the set $\{(Z_i, \vec{r}_i)\}$ of atomic species and positions, and the resulting potential energy E, along with the interatomic forces $\{(\vec{F}_i)\}$. The total potential energy is expressed as the sum of individual atomic energy contributions E_i given by each atomic type's corresponding neural network:

$$E_T = \sum_i E_i \tag{11}$$

Forces are obtained by taking the negative gradient of the potential energy with respect to atomic positions, which ensures that energy conservation is maintained:

$$\vec{F}_i = -\frac{\partial E_T}{\partial \vec{r}_i} \tag{12}$$

Each neural network within the ensemble is initialized with randomly assigned weight parameters θ and trained independently on the same dataset, using a loss function that combines energy and force components through a weighted summation:

$$\mathcal{L}(\theta) = \mathcal{L}_E(\theta) + \lambda \mathcal{L}_F(\theta) \tag{13}$$

Where $\lambda = 0.1$ and $\mathcal{L}_E(\theta), \mathcal{L}_{\mathcal{F}}(\theta)$ are the mean squared error (MSE) losses for the energy and forces, respectively. The hyperparameter configurations used for training the deep ensemble of ANI models are summarized in Table S3.

Table S3: Hyperparameter configurations and performance metrics (RMSE for energy and forces) of the eight neural network models in the deep ensemble trained on the WS22 dataset.

Model No.	Neuron x [72, 64, x, 16]	Activation	Learning rate	RMSE energy (kcal/mol)	RMSE Force (kcal/mol/Å)
1	32	ReLU	0.002	0.391	2.806
2	32	ReLU	0.001	0.161	2.093
3	32	CELU	0.001	0.069	0.851
4	32	CELU	0.002	0.069	0.782
5	64	CELU	0.002	0.046	0.598
6	64	CELU	0.001	0.069	0.759
7	64	ReLU	0.001	0.207	2.162
8	64	ReLU	0.002	0.414	3.059

All other hyperparameters were held constant across the ensemble and are listed in Table S4.

Table S4: Other hyperparameters of ANI models

Model No.	Neuron x<br>[72, 64, x, 16]
Batch Size	128
Max Epochs	1000
Early Stopping Learning Rate	1.0E-7
Learning Rate Reduce Patience	50
Learning Rate Reduce Factor	0.8
Learning Rate Reduce Threshold	0.0
Force Coefficient	0.1
Median Loss	False
Validation Loss Type	Mean RMSE
Fixed Layers	False
Rcr	5.2
Rca	3.5
EtaR	[16.0]
ShfR	[0.9, 1.16875, 1.4375, 1.70625, 1.975, 2.24375, 2.5125, 2.78125, 3.05, 3.31875, 3.5875, 3.85625, 4.125, 4.39375, 4.6625, 4.93125]
Zeta	[32.0]
ShfZ	[0.19635, 0.58905, 0.98175, 1.37445, 1.76715, 2.15984, 2.55254, 2.94524]
EtaA	[8.0]
ShfA	[0.9, 1.55, 2.2, 2.85]

S 4. Post hoc calibration techniques

Predictive uncertainties from both DER and deep ensemble models were found to be miscalibrated, prompting the use of post hoc calibration techniques to improve the alignment between predicted uncertainty and actual prediction errors. To ensure a broad and systematic evaluation, we employed a mix of non-parametric and parametric methods.[4-6]

Isotonic Regression: Isotonic regression (ISR)[4], also referred to as monotonic regression, is a technique for fitting a flexible curve to a set of data points under the constraint that the resulting function is either entirely non-decreasing or non-increasing. Unlike linear regression, it does not require a specific functional form, allowing it to adapt closely to the data as long as the monotonicity condition is met. This makes isotonic regression particularly useful for capturing trends in data where the direction of change is consistent but not necessarily linear. The isotonic regression task can be formulated as a quadratic programming problem

$$y_i = mf(x_i) + \varepsilon \tag{14}$$

where m is an isotonic function. Now, given the training set $\{y_i, f_i\}$, we try to find the isotonic function $\hat{m}$ by minimizing the following equation,

$$\hat{m} = argmin_z \sum \left(y_i - z(f_i)\right)^2 \tag{15}$$

Standard Scaling: Standard scaling[5] (a variance scaling) assumes that errors follow a Gaussian distribution and seeks to align predicted uncertainty distributions accordingly. To achieve this, a single scaling factor is applied to the predicted standard deviations. This factor is learned by minimizing a chosen calibration loss function, often the miscalibration area, so that the predicted uncertainty becomes more consistent with the observed errors. Although this method is more constrained compared to non-parametric approaches, such as isotonic regression, it maintains the Gaussian shape of the uncertainty distribution. These two methods are computationally efficient, require no model retraining, and offer flexibility across architectures, making them appropriate for a comparative study like ours.

GP-Normal: GP-Normal[6] is a parametric recalibration method that extends temperature scaling by modelling the variance adjustment as a function of the input features using a Gaussian Process (GP). This approach enables the predicted variances to vary with the input while keeping the predicted mean unchanged. GP-Normal retains a Gaussian parametric structure and calibrates variance by aligning predicted values with observed squared errors. One key benefit of GP-Normal is its ability to learn smooth and adaptive mappings within a

Bayesian framework, making it suitable for capturing complex relationships between prediction errors and uncertainty.

To ensure a broad and balanced evaluation, we combine GP-Normal with both isotonic regression and standard scaling. These methods span a range of assumptions and model capacities, from flexible parametric approaches to non-parametric baselines. This variety enables a systematic analysis of how different recalibration strategies affect the trustworthiness of uncertainty estimates.

S 5. Post hoc calibration results of EGNN-DER

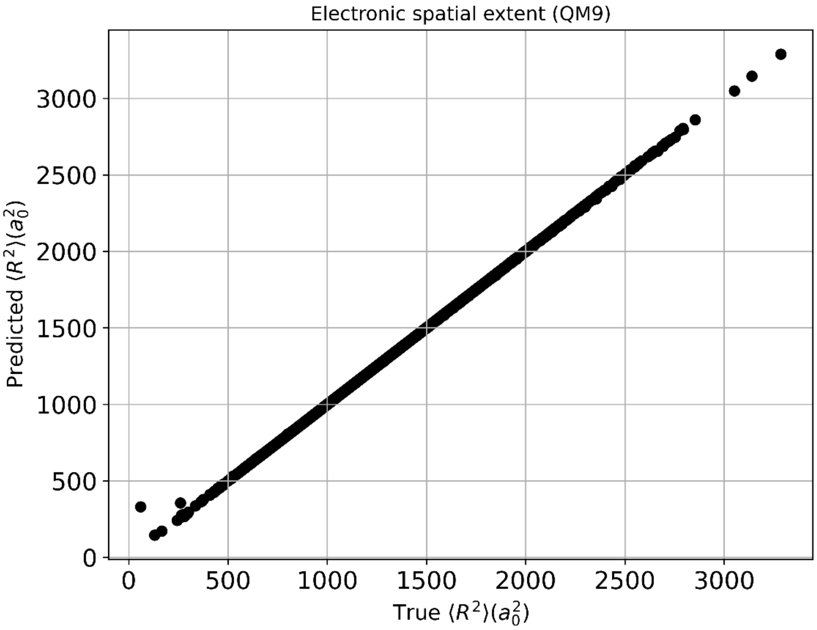

Figure S1: Predicted vs. true values of the electronic spatial extent (a_o^2) for the test set of QM9 dataset using EGNN-DER.

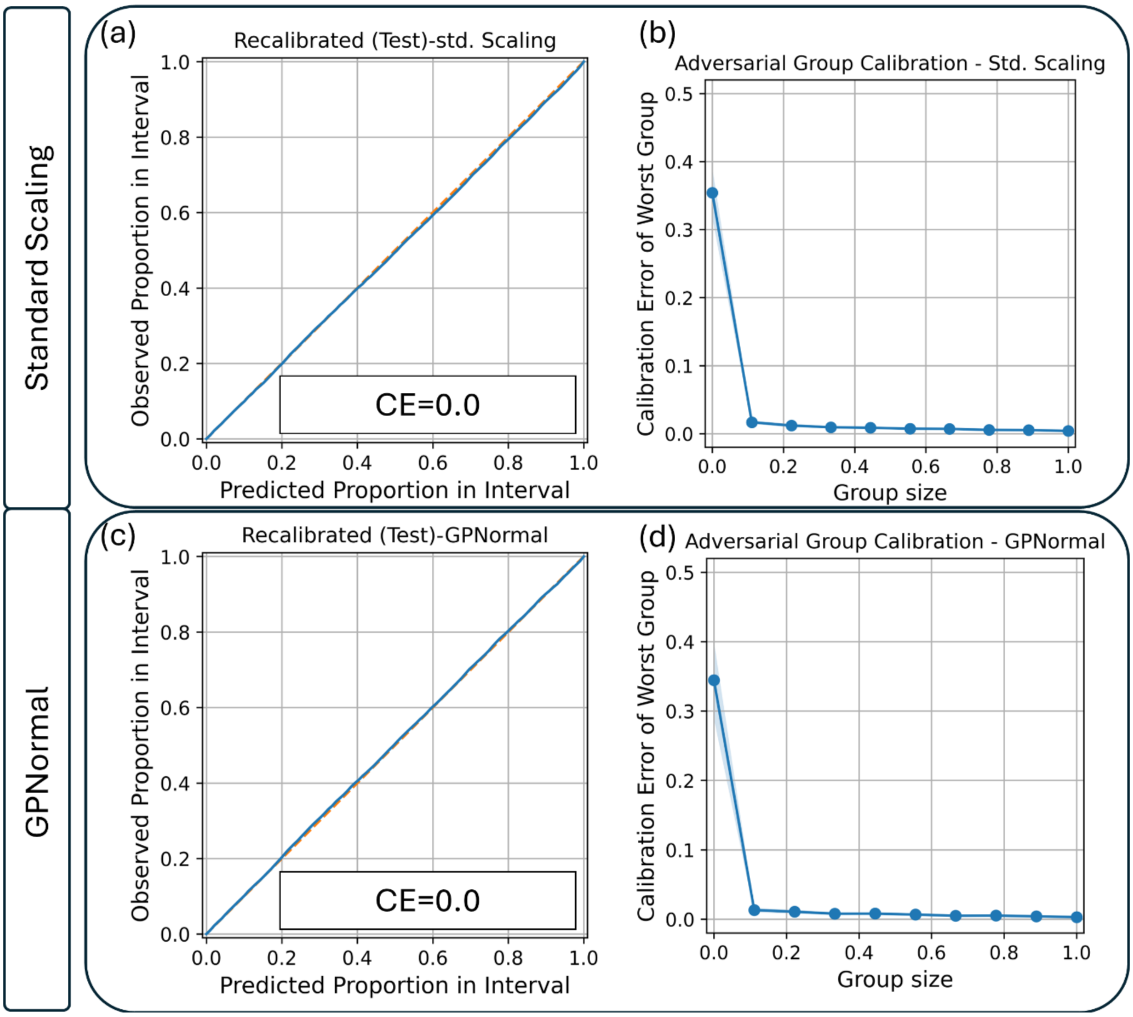

Figure S2: Uncertainty calibration and adversarial group calibration plots for predicting electronic spatial extent ($\langle R^2 \rangle$) in the QM9 dataset using two recalibration methods: (a, b) Standard scaling (non-parametric method): Perfect calibration is observed with a miscalibration area of 0.00, and adversarial group calibration shows minimal errors across group sizes. (c, d) GPNormal (parametric method): Similarly, perfect calibration is achieved with a calibration error of 0.00, and adversarial group calibration indicates minimal errors across group sizes.

S 6. Energy Uncertainty Calibration in WS22

This section presents calibration results for predicted energy uncertainties on the WS22 acrolein dataset. It includes initial miscalibration curves and comparisons of three post hoc calibration techniques: Isotonic Regression (ISR), Standard Scaling, and GP-Normal.

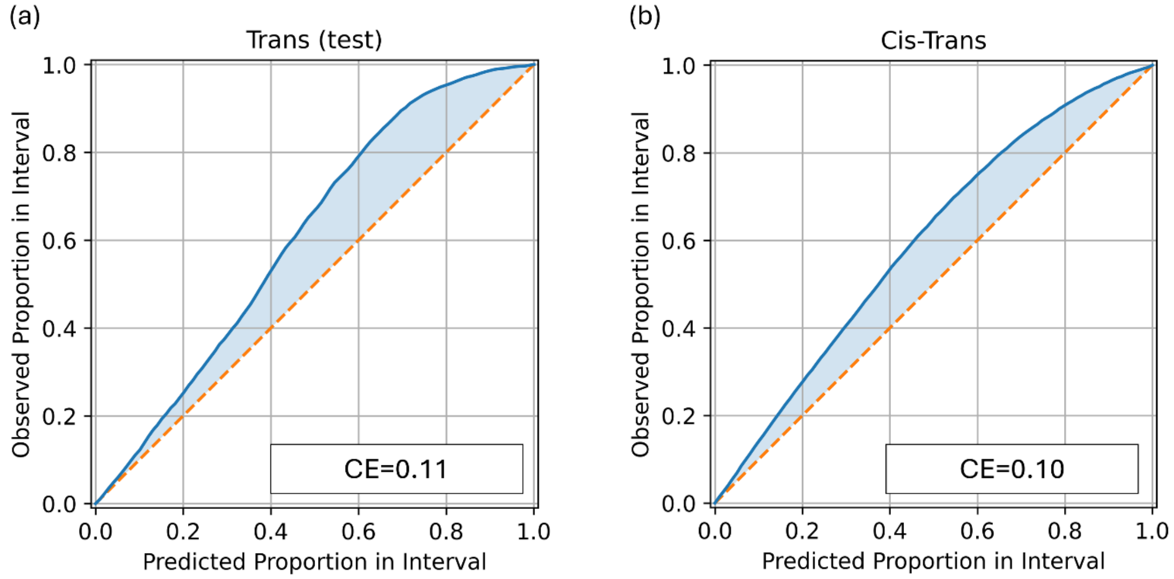

Figure S3: (a) Energy uncertainty miscalibration curve for Trans (test). The observed proportion versus predicted proportion shows a slight deviation from perfect calibration, with a CE of 0.11. (b) Cis-Trans (geodesic interpolation); similar miscalibration is observed, with a CE of 0.10.

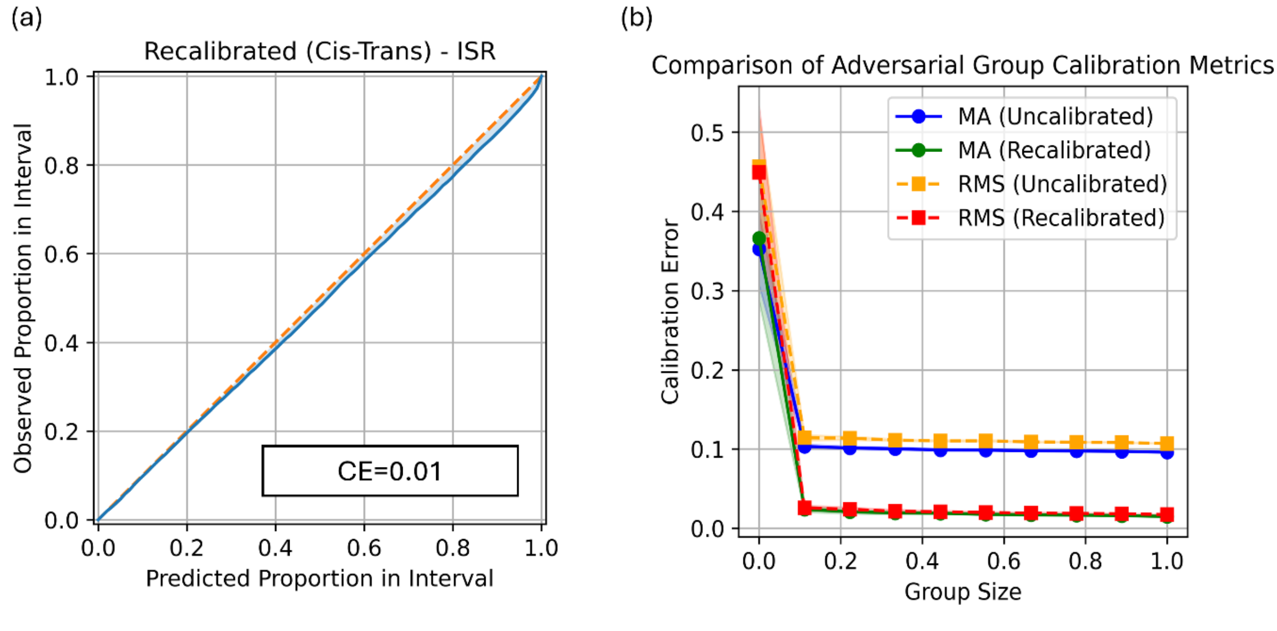

Figure S4: (a) Post hoc recalibration for the Cis-Trans region using ISR achieves near-perfect calibration with a CE of 0.01, indicating highly accurate uncertainty estimates. (b) Adversarial group calibration metrics compare miscalibration area (MA) and root mean square (RMS) errors for uncalibrated and recalibrated models. The recalibrated model exhibits very low miscalibration across all group sizes, including smaller groups, demonstrating a high level of calibration accuracy.

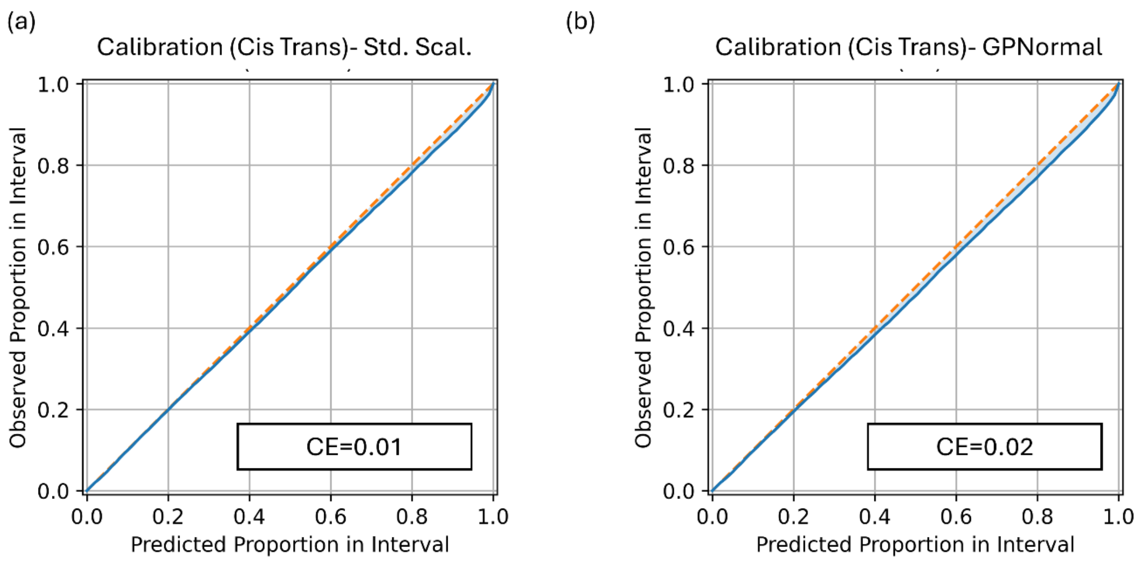

Figure S5: Post hoc calibration results for the Cis-Trans region: (a) Standard Scaling achieves a CE of 0.01, demonstrating excellent calibration. (b) GPNormal produces similar results with a slightly higher CE of 0.02. Both methods show comparable performance, reinforcing the effectiveness of post hoc calibration.

S 7. Justification for Using Force Norm in Uncertainty Calibration

Simulations rely on individual force vectors, but in this work, we evaluate the **force norm** to represent the uncertainty associated with force predictions. This choice is motivated by:

1. **Tractability**: The force norm is a scalar quantity, which aligns with the scalar-focused calibration techniques employed throughout this study (e.g., isotonic regression, GP-Normal, standard scaling).
2. **Proxy for Directional Reliability**: The force norm can serve as a surrogate for directional agreement between predicted and true forces. In Figure S6 we show that agreement in norms often corresponds to well-aligned component vectors. Conversely, mismatches in norm often indicate angular disagreement.

To validate this, we performed a quantitative comparison in Error! Reference source not found., where we computed a directional disagreement metric defined as $1-|\cos(\theta)|$, with θ being the angle between predicted and reference force vectors. We observed a strong correlation between this directional disagreement and the predicted force norm uncertainty, supporting the interpretation that norm-based uncertainty effectively reflects both **magnitude** and **directional** reliability.

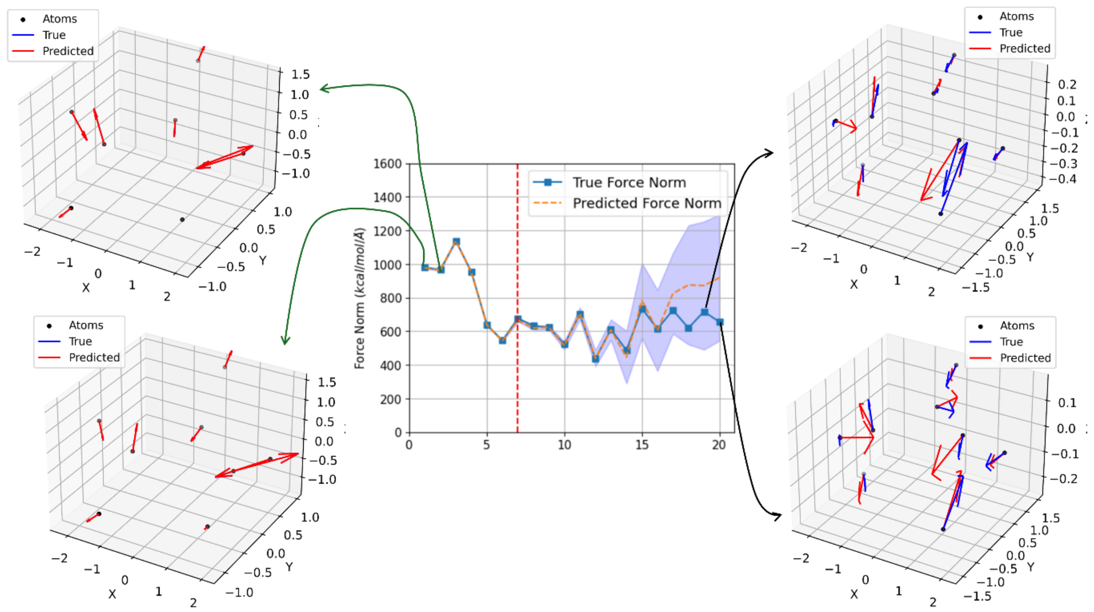

Figure S6: Comparison of predicted and true force vectors at selected points along a geodesic path. The central panel shows force norm evolution and uncertainty across the path, with insets highlighting individual geometries. Good agreement in force norm evolution and uncertainty across the path, with insets highlighting individual geometries. Good agreement in force norms corresponds to close alignment of vector components (left), while divergence in norms reflects noticeable directional mismatches (right).

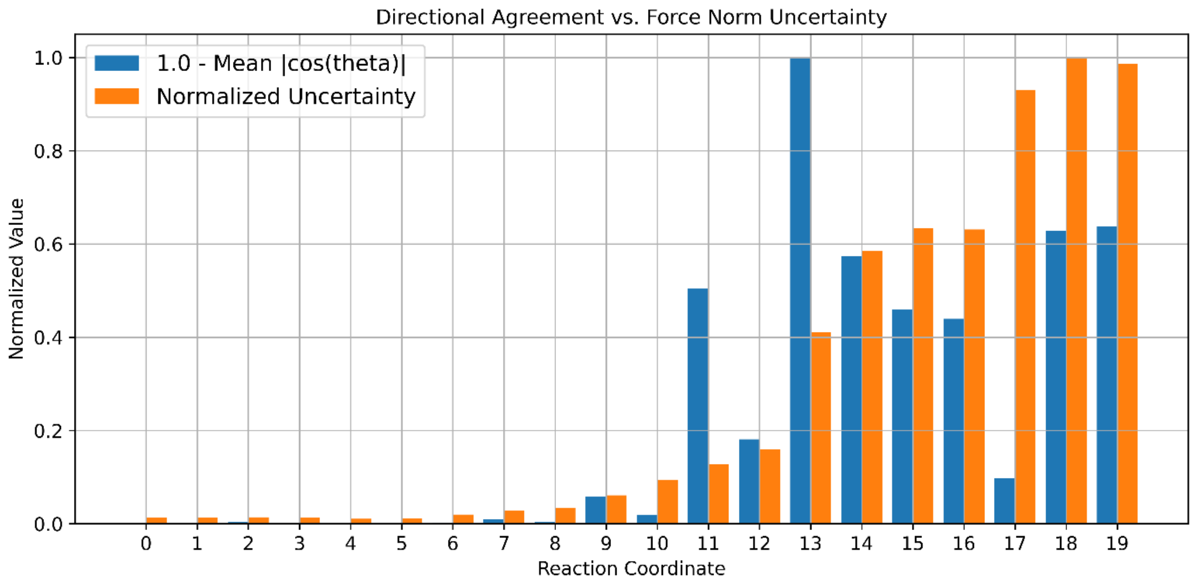

Figure S7: Comparison of directional error and force norm uncertainty along the cis–trans isomerization pathway. The plot shows the normalized directional disagreement (1 − |cos(θ)|) between predicted and true force vectors, alongside normalized predicted force norm uncertainty. Both metrics consistently highlight regions with poor model performance. The strong correlation indicates that force norm uncertainty captures not only scalar magnitude deviation but also directional errors in force prediction, supporting its use as a proxy for per-component reliability in scalable uncertainty quantification frameworks.

S 8. Calibration of Force Uncertainty Predictions

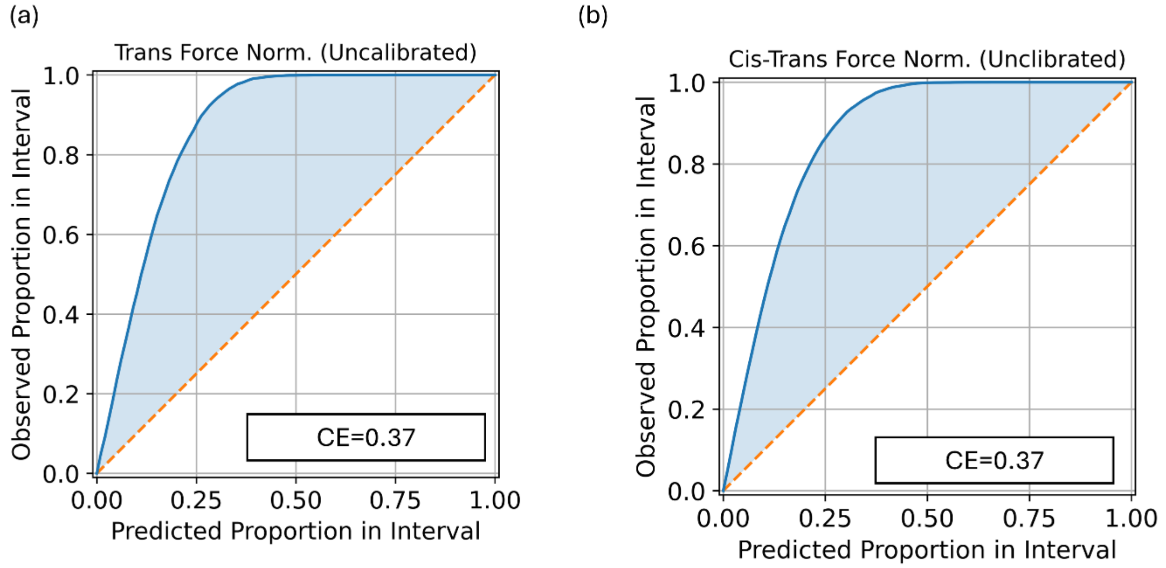

Figure S8: Miscalibration curves for force uncertainty predictions before recalibration. (a) Test set (trans) and (b) cis–trans interpolation region. The curves show significant underconfidence in both cases, with a calibration error (CE) of 0.37.

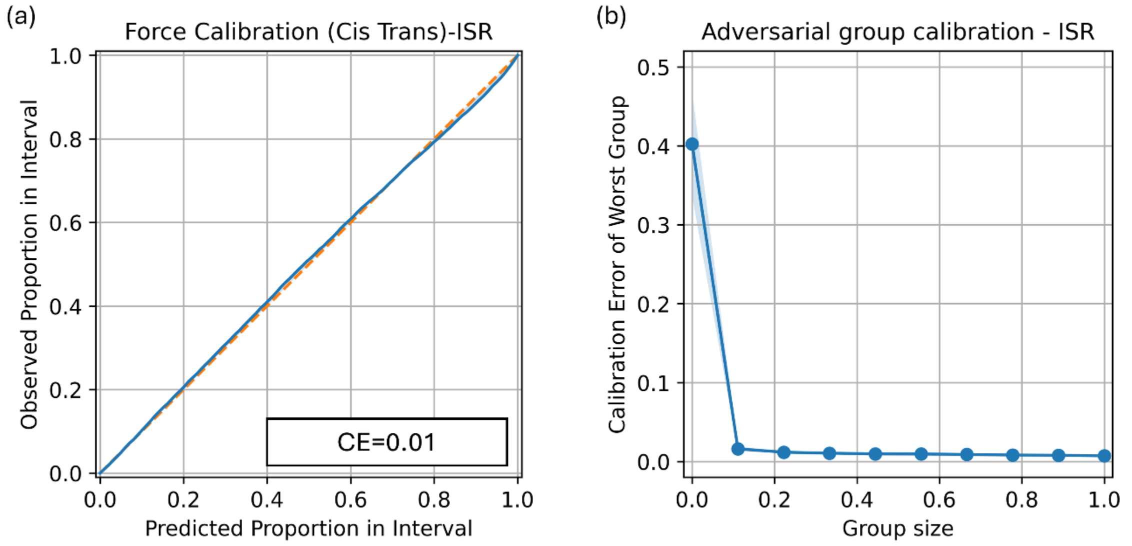

Figure S9: (a) Results after post hoc calibration of force uncertainty using isotonic regression (ISR). (b) Adversarial group calibration (AGC) indicates consistent reliability across group sizes.

S 9. Force Uncertainty Thresholding Across Geodesic Paths

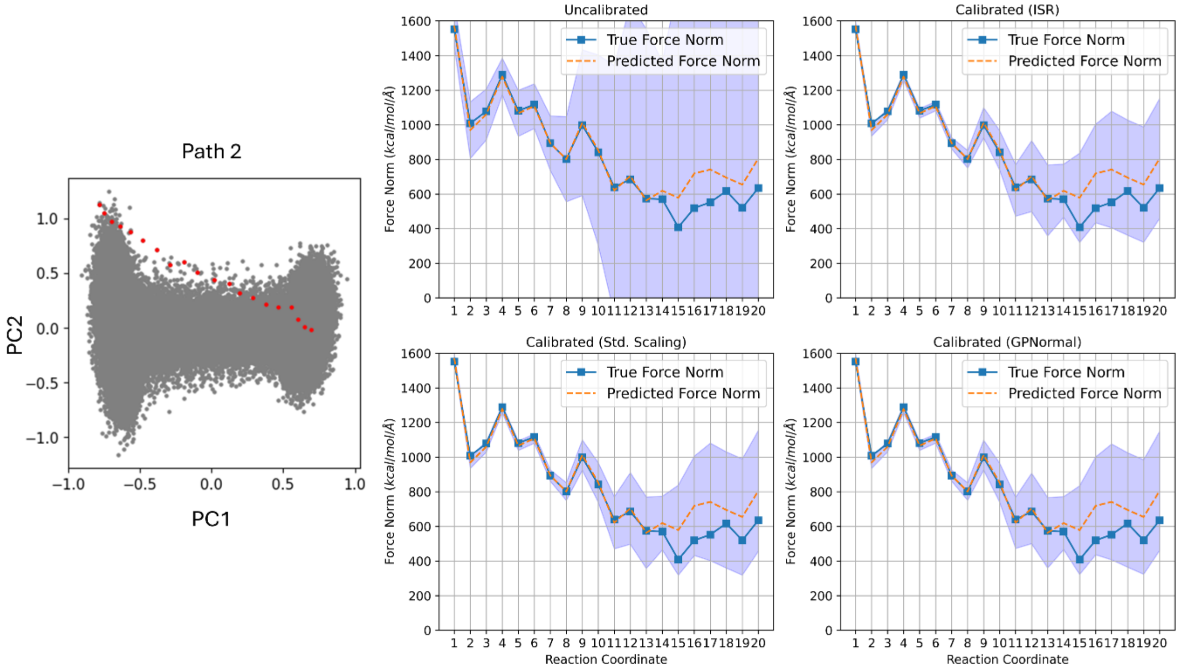

Figure S10: Force prediction uncertainties ($2\sigma_i^F$) and true force norms along **Path 2** (from Geodesic interpolation).

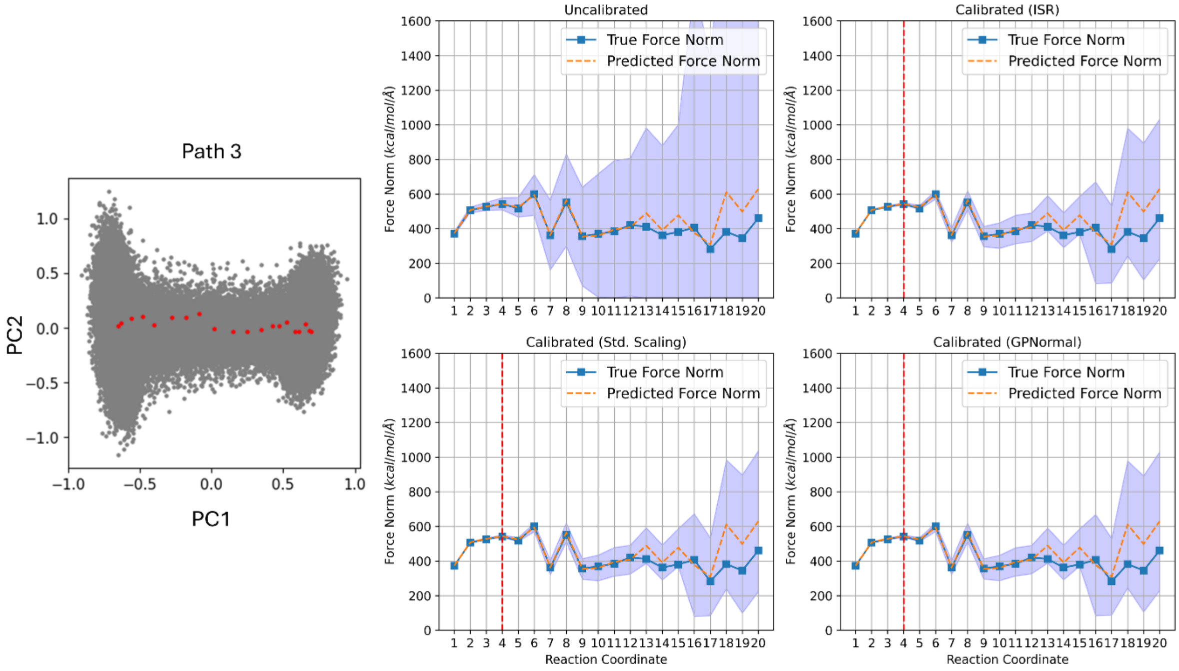

Figure S11: Force prediction uncertainties ($2\sigma_i^F$) and true force norms along **Path 3** derived from geodesic interpolation. After calibration, the uncertainty threshold of 10 kcal/mol/Å is only exceeded beyond point 4, allowing the first 4 points to be excluded from query—thus saving redundant computations at the early stages of the path.

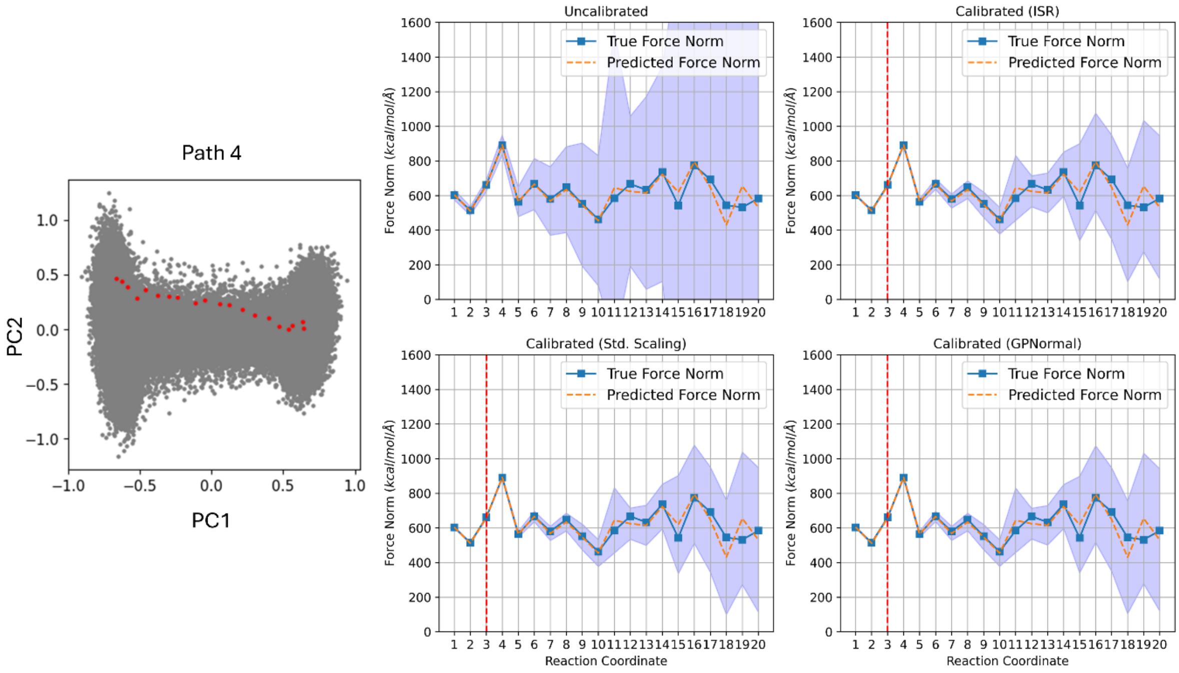

Figure S12: Force prediction uncertainties ($2\sigma_i^F$) and true force norms along **Path 4** derived from geodesic interpolation. After calibration, the uncertainty threshold of 10 kcal/mol/Å is only exceeded beyond point 3, allowing the first 3 points to be excluded from query—thus saving redundant computations at the early stages of the path.

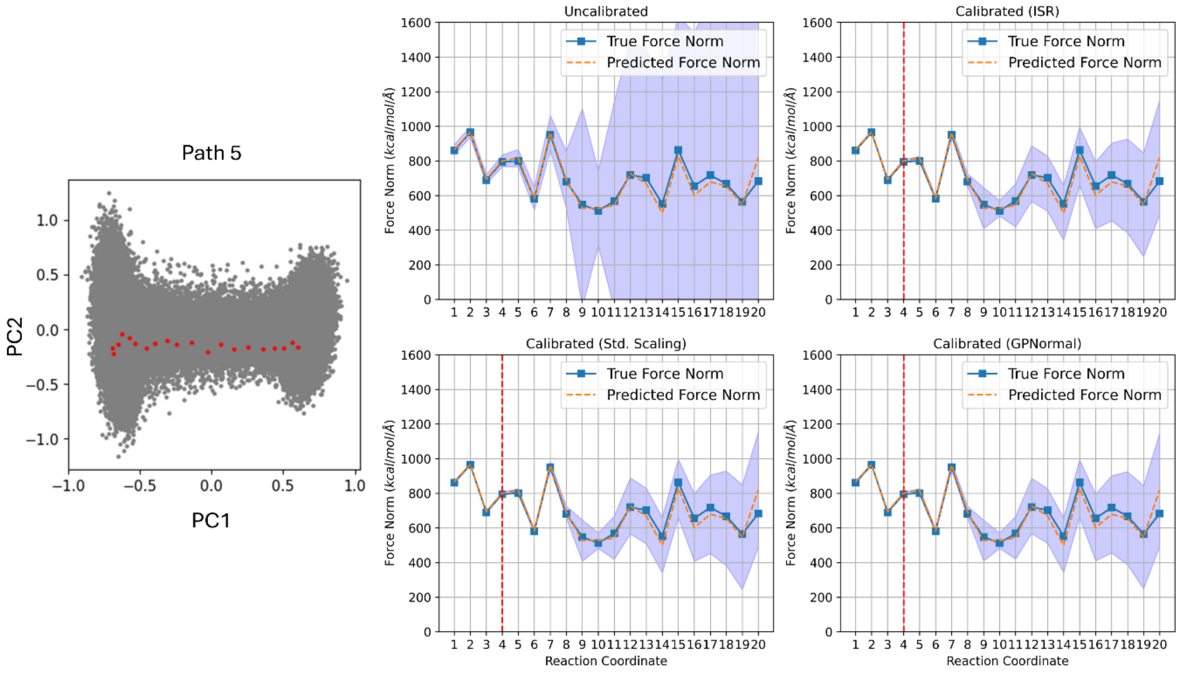

Figure S13: Force prediction uncertainties ($2\sigma_i^F$) and true force norms along **Path 5** derived from geodesic interpolation. After calibration, the uncertainty threshold of 10 kcal/mol/Å is only exceeded beyond point 4, allowing the first 4 points to be excluded from query—thus saving redundant computations at the early stages of the path.

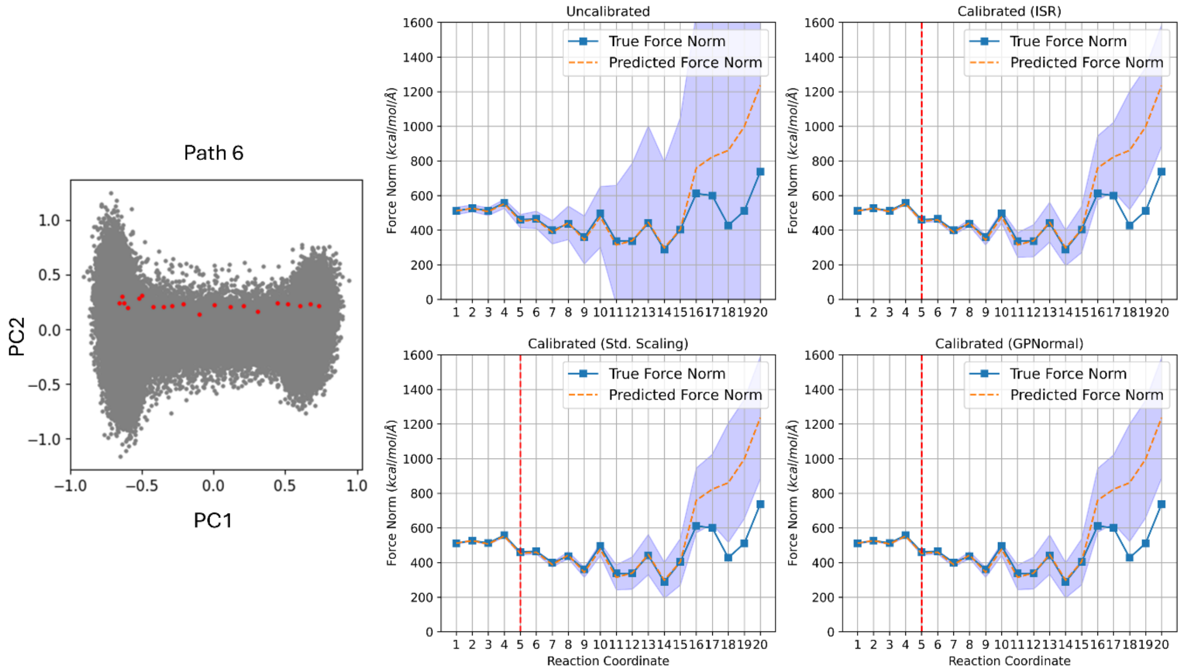

Figure S14: Force prediction uncertainties ($2\sigma_i^F$) and true force norms along **Path 6** derived from geodesic interpolation. After calibration, the uncertainty threshold of 10 kcal/mol/Å is only exceeded beyond point 5, allowing the first 5 points to be excluded from query—thus saving redundant computations at the early stages of the path.

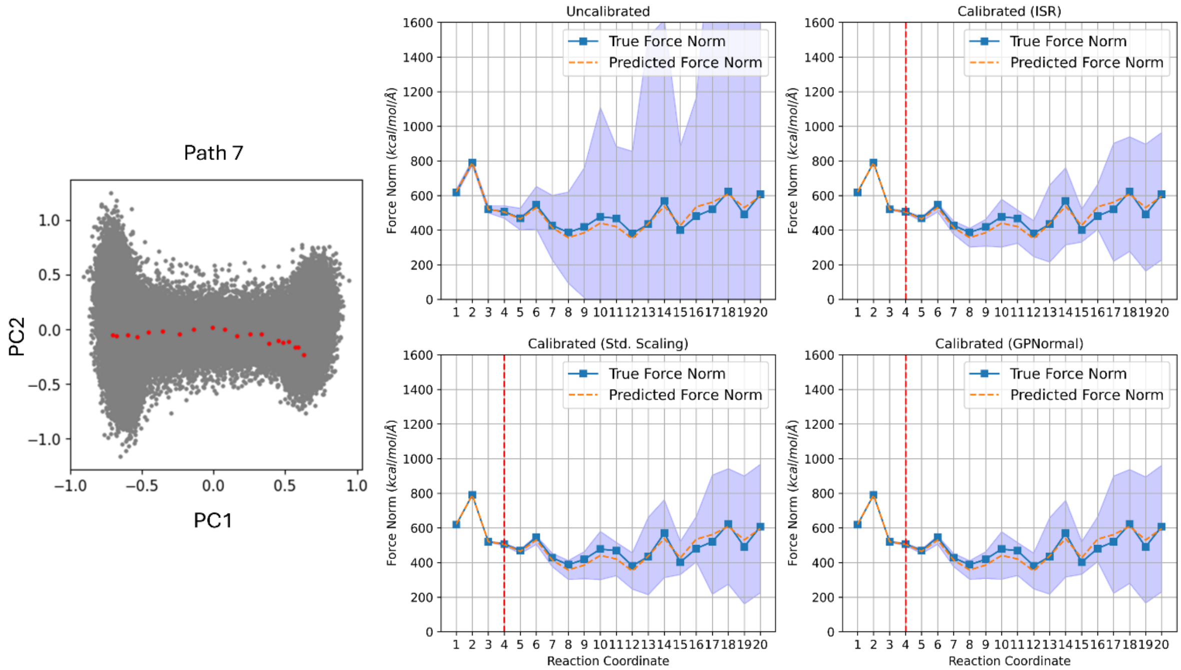

Figure S15: Force prediction uncertainties ($2\sigma_i^F$) and true force norms along **Path 7** derived from geodesic interpolation. After calibration, the uncertainty threshold of 10 kcal/mol/Å is only exceeded beyond point 4, allowing the first 4 points to be excluded from query—thus saving redundant computations at the early stages of the path.

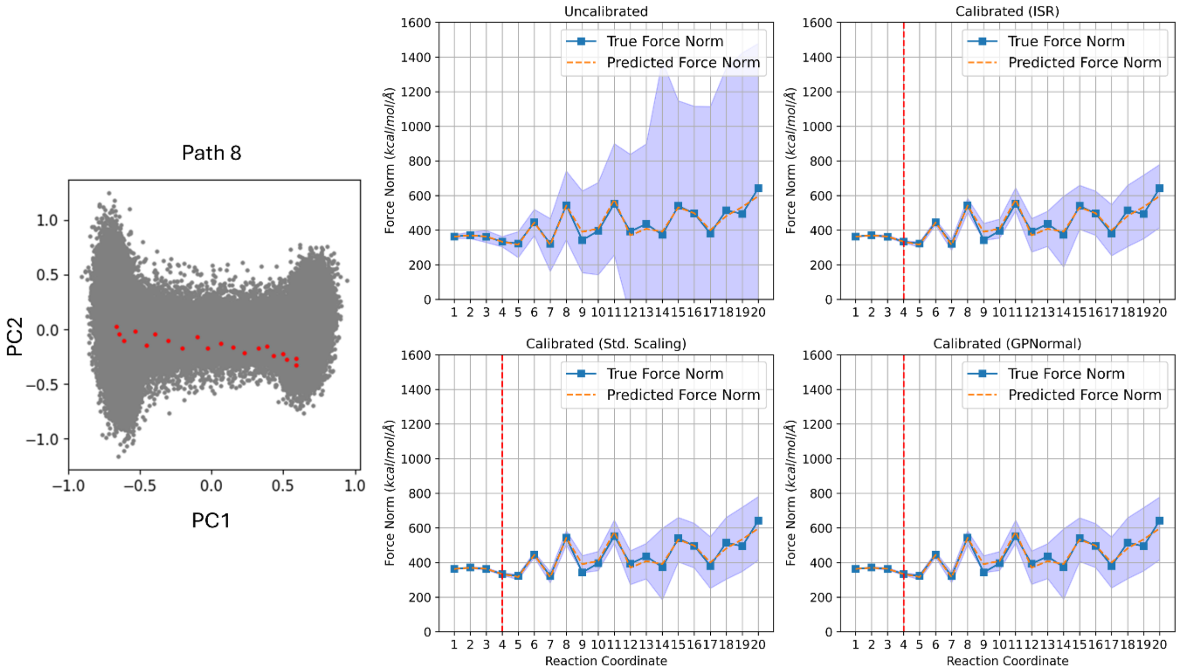

Figure S16: Force prediction uncertainties ($2\sigma_i^F$) and true force norms along **Path 8** derived from geodesic interpolation. After calibration, the uncertainty threshold of 10 kcal/mol/Å is only exceeded beyond point 4, allowing the first 4 points to be excluded from query—thus saving redundant computations at the early stages of the path.

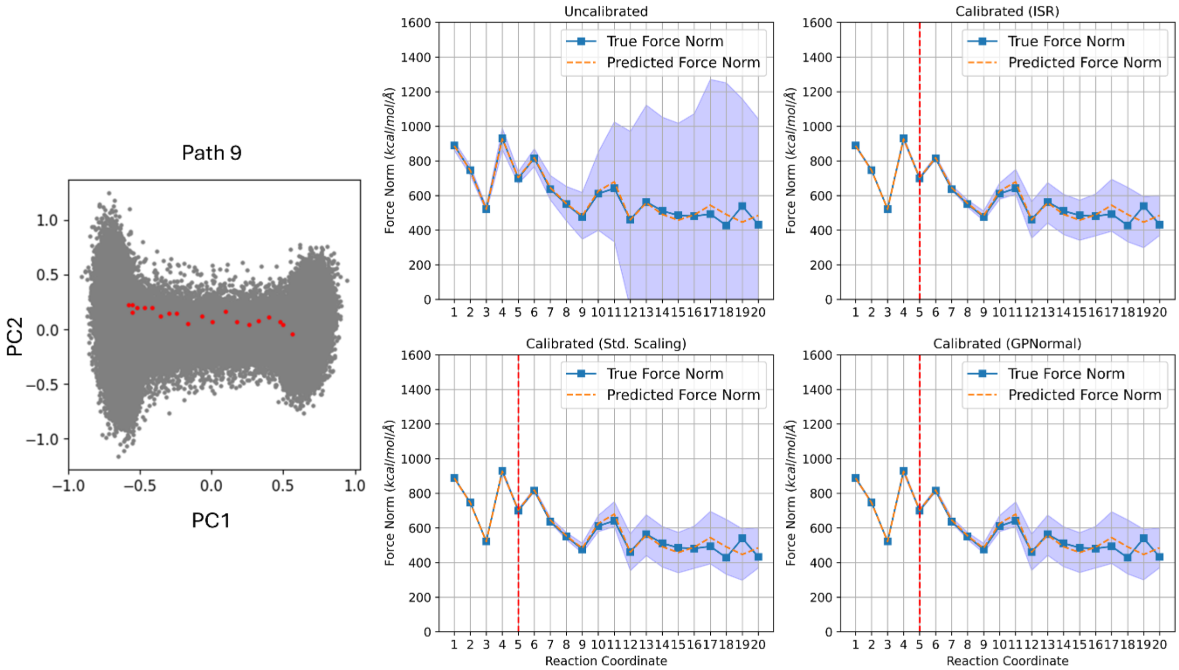

Figure S17: Force prediction uncertainties ($2\sigma_i^F$) and true force norms along **Path 9** derived from geodesic interpolation. After calibration, the uncertainty threshold of 10 kcal/mol/Å is only exceeded beyond point 5, allowing the first 5 points to be excluded from query—thus saving redundant computations at the early stages of the path.

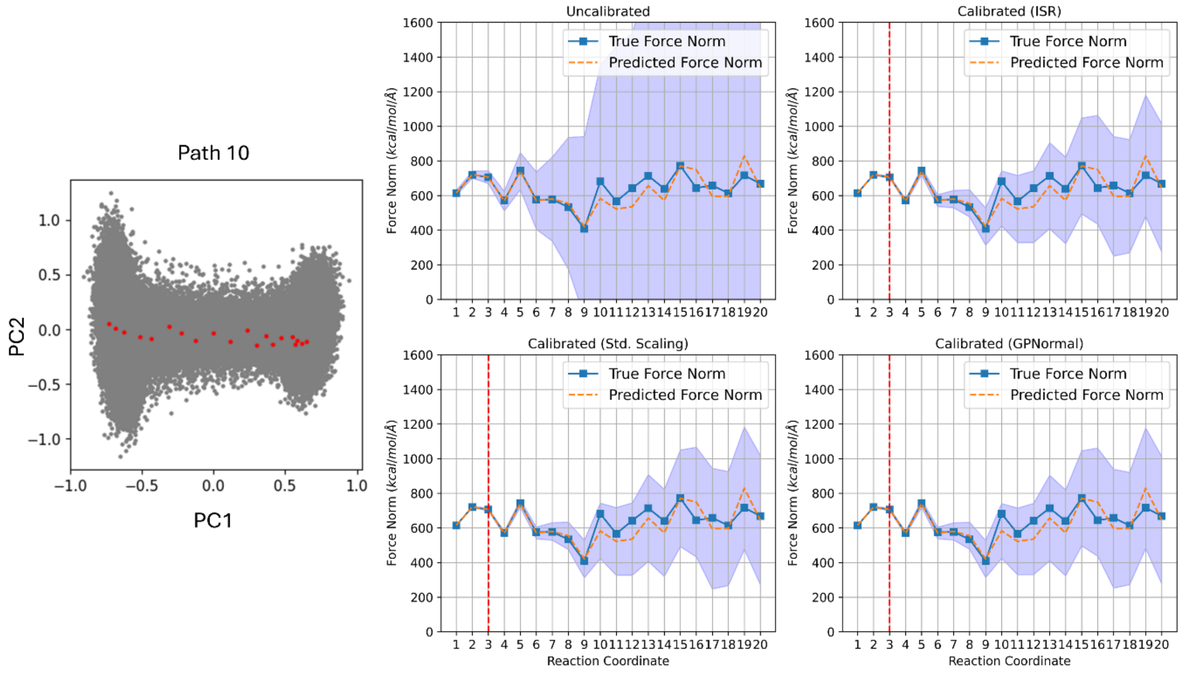

Figure S18: Force prediction uncertainties ($2\sigma_i^F$) and true force norms along **Path 10** derived from geodesic interpolation. After calibration, the uncertainty threshold of 10 kcal/mol/Å is only exceeded beyond point 3, allowing the first 3 points to be excluded from the query—thus saving redundant computations at the early stages of the path.

S 10. References

[1] K. Nehil-Puleo, C. D. Quach, N. C. Craven, C. McCabe, P. T. Cummings *J. Phys. Chem. B*. **2024**, *128*, 1108-1117.

[2] A. Amini, W. Schwarting, A. Soleimany, D. Rus *Adv. Neural Inf. Process Syst.* **2020**, *33*, 14927-14937.

[3] L. I. Vazquez-Salazar, E. D. Boittier, M. Meuwly *Chem. Sci.* **2022**, *13*, 13068-13084.

[4] V. Kuleshov, N. Fenner, S. Ermon, in *Proc. 35th Int. Conf. on Machine Learning (ICML 2018)*, Vol. 80 (Eds.: J. G. Dy, A. Krause), PMLR, 2018, pp. 2801–2809.

[5] D. Levi, L. Gispan, N. Giladi, E. Fetaya, *Sensors* **2022**, *22*, 5540.

[6] H. Song, T. Diethe, M. Kull, P. Flach, *Proc. Mach. Learn. Res.* **2019**, *97*, 5897–5906.
